# Elevated H3K4me3 Through MLL2-WDR82 upon Hyperglycemia Causes Jagged Ligand Dependent Notch Activation to Interplay with Differentiation State of Endothelial Cells

**DOI:** 10.3389/fcell.2022.839109

**Published:** 2022-03-22

**Authors:** Niyati Pandya Thakkar, Beatriz Maria Veloso Pereira, Yash T. Katakia, Shyam Kumar Ramakrishnan, Sumukh Thakar, Ashima Sakhuja, Gayathry Rajeev, S. Soorya, Karina Thieme, Syamantak Majumder

**Affiliations:** ^1^ Department of Biological Sciences, Birla Institute of Technology and Science (BITS), Pilani Campus, Pilani, India; ^2^ Laboratório de Bases Celulares e Moleculares da Fisiologia Renal, Departamento de Fisiologia e Biofísica, Instituto de Ciências Biomédicas, Universidade de Sao Paulo, Sao Paulo, Brazil

**Keywords:** EndMT, epigenetics, H3K4Me3, hyperglycemia, MLL2 and notch signaling

## Abstract

Endothelial-to-mesenchymal transition (EndMT) is a hallmark of diabetes-associated vascular complications. Epigenetic mechanisms emerged as one of the key pathways to regulate diabetes-associated complications. In the current study, we aimed to determine how abrupt changes in histone 3 lysine 4 tri-methylation (H3K4me3) upon hyperglycemia exposure reprograms endothelial cells to undergo EndMT. Through *in vitro* studies, we first establish that intermittent high-glucose exposure to EC most potently induced partial mesenchyme-like characteristics compared with transient or constant high-glucose-challenged endothelial cells. In addition, glomerular endothelial cells of BTBR Ob/Ob mice also exhibited mesenchymal-like characteristics. Intermittent hyperglycemia-dependent induction of partial mesenchyme-like phenotype of endothelial cells coincided with an increase in H3K4me3 level in both macro- and micro-vascular EC due to selective increase in MLL2 and WDR82 protein of SET1/COMPASS complex. Such an endothelial-specific heightened H3K4me3 level was also detected in intermittent high-glucose-exposed rat aorta and in kidney glomeruli of Ob/Ob mice. Elevated H3K4me3 enriched in the promoter regions of Notch ligands Jagged1 and Jagged2, thus causing abrupt expression of these ligands and concomitant activation of Notch signaling upon intermittent hyperglycemia challenge. Pharmacological inhibition and/or knockdown of MLL2 in cells *in vitro* or in tissues *ex vivo* normalized intermittent high-glucose-mediated increase in H3K4me3 level and further reversed Jagged1 and Jagged2 expression, Notch activation and further attenuated acquisition of partial mesenchyme-like phenotype of endothelial cells. In summary, the present study identifies a crucial role of histone methylation in hyperglycemia-dependent reprograming of endothelial cells to undergo mesenchymal transition and indicated that epigenetic pathways contribute to diabetes-associated vascular complications.

## Introduction

Hyperglycemia is the primary indicator of diabetes and has long been used as a measure to monitor the progression of diabetes. Hyperglycemia is reported to generate endothelial dysfunction at a systemic level, and repercussions include end-stage vascular complications. The vascular system is heterogeneous and versatile, tremendously sensitive to environmental stimuli, with efficient response to organ demands. It plays an elemental role in maintaining homeostasis and systemic circulation; any damage elevates systemic ramifications (Deanfield et al., 2007; Avogaro et al., 2011; Reiterer and Branco, 2020). With chronic exposure to hyperglycemia, the endothelium exhibits a chain of intracellular events promoting endothelial dysfunction. It is well-accepted that endothelial dysfunction precedes the development of atherosclerosis, a complication commonly associated with diabetes. Endothelial dysfunction is responsible for many microvascular complications associated with diabetes, including nephropathy, retinopathy, and neuropathy, whereas the macrovascular complications manifest as accelerated atherosclerosis, resulting in premature ischemic heart disease, heightened risk of cerebrovascular disease, and severe peripheral vascular disease (Clark and Lee, 1995; Feener and King, 1997).

In the experimental diabetes rodent model, hypertrophy of the mesenteric arteries (Cooper et al., 1994) and increase in blood vessel size is due to surplus smooth muscle cells in the medial layer of the vessel (Vranes et al., 1999). Although proliferation and migration of medial smooth muscle cells to the intima layer (Clowes and Schwartz, 1985) is pivotal in such a pathological context, recent findings hint an alternative mechanism through trans-differentiation of existing endothelial cells (EC) to mesenchymal cells, a phenomenon termed endothelial-to-mesenchymal transition (EndMT) (Zeisberg et al., 2007). TGF-β appears to be an important mediator of vascular injury, and numerous pathogenic stimuli are involved in the upregulation of TGF-β, including glucose-induced PKC activation, advanced glycation products, and Ang II (Rumble et al., 1997; Rumble et al., 1998). Induction of TGF-β in EC results in a mesenchymal phenotype while upregulated levels of TGF-β are consistently reported in atherosclerotic lesions and are associated with plaque instability (Chen et al., 2015; Evrard et al., 2016). Furthermore, studies show atypical activation of Notch signaling in ECs displays a phenotype like EndMT (Noseda et al., 2004; Liu et al., 2014). The Notch pathway is a highly conserved intercellular signaling mechanism, activated with the binding of transmembrane ligands of the Jagged (Jagged1 and Jagged2) and Delta (Delta-like 1 [Dll-1], Dll3, and Dll4) families with Notch receptors (Notch1 to -4) expressed on adjacent cells (trans-interaction) (Artavanis-Tsakonas et al., 1999; Nichols et al., 2007) or same cells (cis-interaction) (Jacobsen et al., 1998; Sprinzak et al., 2010). Studies demonstrate that expression of the mesenchyme-specific protein Slug is directly activated by Notch in EC and Slug expression is required for Notch-mediated repression of the vascular endothelial cadherin promoter, which in turn promotes migration of transformed endothelial cells (Niessen et al., 2008). Further reports show that Notch1-Jagged1 interaction and subsequent activation of Notch signaling results in upregulated expression of α-smooth muscle actin (αSMA), a well-accepted marker of smooth muscle cells (Noseda et al., 2006). Work from our lab indicates that activation of Notch signaling in EC upon inflammatory switch is governed by epigenetic mechanisms that involve a dynamic alteration of an activating H3K4me3 and a repressing H3K27me3 mark in Notch gene promoters (Katakia et al., 2021). This was partly mediated by an alteration in H3K27me3-specific methyltransferase EZH2 localization and an increase in demethylases JMJD3 and UTX. Studies describe that loss of EZH2 expression is associated with reduced H3K27me3 marks at the TAGLN (transgelin [SM22α]) promoter, resulting in the transcriptional activation of TAGLN (SM22α), a mesenchymal gene that facilitates the process of EndMT (Maleszewska et al., 2015). Endothelial identity and function are critically controlled by the histone demethylase JMJD2B by regulating the level of H3K9me3, which is induced by EndMT-promoting, proinflammatory, and hypoxic conditions, and supports acquiring a mesenchymal phenotype (Glaser et al., 2020). Histone H4 acetylation is involved in the synergistic upregulation of specific SMAD3 target genes (HEY1 and ANKRD1) upon TGF-β1 stimulation and Notch activation (Fu et al., 2009).

Diabetes-dependent pathogenesis may be driven by “metabolic memory” (Brasacchio et al., 2009) in which, upon a brief encounter with hyperglycemia, the epigenetic landscape of fully differentiated cells alters, leading to cellular dysfunction contributing to pathogenesis of a disease. The epigenetic landscape of ECs exposed to an intermittent high glucose (often termed “oscillating high glucose level”) condition remains to be entirely explored. Indeed, diabetic patients experience “hyperglycaemic spikes” (Hanssen et al., 2020), which hints that intermittent hyperglycemia is more deleterious than constant hyperglycaemic conditions. A study trial in a group of diabetic patients suggests that oxidative stress plays a major role in damaging the endothelium, a phenomenon profound in oscillatory high-glucose conditions (Ceriello et al., 2008). Molecular studies on EC subjected to high glucose in a pulsatile fashion displayed increased ROS production *via* PKC-dependent activation of NAD(P)H oxidase, leading to increased cell apoptosis (Quagliaro et al., 2003). Previous studies from our lab report deposition of H3K27me3 marks in human umbilical vein endothelial cells (HUVEC) exposed to intermittent hyperglycemia caused by a reduction in EZH2 threonine 367 phosphorylation and nuclear retention of EZH2. Heightened H3K27me3 on *KLF2* and *KLF4* gene promoters results in its repression, aiding in endothelial inflammation (Thakar et al., 2021). In the present study, we report acquisition of an epigenetically activating H3K4me3 mark on gene promoters of Notch ligands *Jagged1* and *Jagged2*, thereby causing their expression in EC subject to intermittent high-glucose conditions resulting in a partial mesenchyme-like phenotype of EC.

## Materials and Methods

### Cell Culture and Treatment

HUVEC (#CL002-2XT25, Himedia) were cultured in HiEndoXL^™^ Endothelial Cell Expansion medium (#AL517; Himedia) supplemented with 4% endothelial growth supplement, 3% fetal bovine serum (#RM1112, FBS; Himedia), and 1X penicillin streptomycin glutamine (#10378, PS; Gibco). Cells were incubated at 37°C with a constant 5% CO_2_ condition. HUVEC from passages 4–9 were used to perform all the experiments. HUVEC were given at least 48 h to synchronize and reach certain confluence prior to applying any treatment conditions. Human primary kidney glomerular endothelial cells (HGMEC) (#H-6014G; Cell Biologics) were cultured in complete human endothelial cell medium (#H1168; Cell Biologics). HGMEC from passages 4–6 were used for all the reported data. Upon receiving both HUVEC and HGMEC from the respective supplier, we tested the purity of these cells through immunofluorescence analysis by immuno-staining the cells with CD31 and/or CD144 antibody followed by imaging to identify positive cells. During IHG treatment, both HUVEC and HGMEC were kept in low-growth-factor– and low-serum–containing media. For treatment, cells were seeded in a six-well plate with count of 1 × 10^5^ cells per well. The IHG, THG, and CHG treatment groups were exposed to a 3-day, 25 mM glucose treatment medium for 12-h pulsatile intervals, early 24 h, and constant 72 h duration, respectively.

### Mouse Kidney Immunofluorescence Staining

All the experimental protocols were conducted in accordance with the guidelines of the Brazilian College for Animal Experimentation and were approved by the Institutional Animal Care and Research Advisory Committee (CEUA #5348280918). Male diabetic and obese mice (Ob/Ob) (BTBR.Cg-Lepob/WiscJ; The Jackson laboratory) on a BTBR background and wild-type (WT) mice were employed in this study. Mice were weaned at 21 days of age and genotyped following the protocol provided by The Jackson Laboratory. Animals were maintained in the Pharmacology Department Animal Care Facility, University of Sao Paulo, Sao Paulo, SP, Brazil. Animal were housed in cages (three to four animals/cage) and maintained at a 12:12 h light–dark cycle and 23°C ± 2°C with free access to standard diet (Nuvilab-Nuvital Nutrients Ltd., Parana, Brazil). Mouse kidney sections from WT and Ob/Ob male mice, 14 weeks old, were formalin-fixed and paraffin-embedded, followed by heat-induced epitope retrieval with citrate buffer. The sections were blocked (#X909, Protein Block; Dako), and incubated overnight at 4°C with the first primary antibody. On the subsequent day, after repeated washes with TBS-T, sections were incubated with secondary antibody for 2 h. Next, the second first antibody was added, and sections were incubated for 2 h at room temperature, followed by incubation with the second secondary antibody for another 2 h. DAPI was used at a concentration of 1:20,000. Images were either taken at 60X in the Microscopy Nikon Eclipse E600 with an Olympus DP72 camera or taken at 40X in the Zeiss LSM 780 Microscope with ZEN 2011 SP7 FP3 (black) software version 14.0.21.201. The antibodies used are specified in Supplementary Table S1. For colocalization analysis of CD31 and α-SMA–stained kidney tissues, we utilized the plugin of ImageJ named Coloc2, which analyzes different parameters of colocalization, including the Pearson correlation coefficient, indicating the degree of overlap between two fluorescent labeled proteins. Colocalization analyses were performed by selecting only the glomerular area of the tissue, and therefore, Pearson correlation values indicate the level of colocalization in the glomerular part of the kidney sections.

### Animal Dissection and Treatment Conditions

All experimental procedures for the rodent studies were approved by the Institutional Animal Ethics Committee of BITS Pilani, Pilani Campus. Male Wistar rats aged 12–16 weeks were selected for the *ex vivo* experiment. The rats were fed on a normal chow diet. Rats were anesthetized and dissected by an incision from the ventral end. The hearts and aortas were perfused with PBS to remove any blood cells from the vessels. The primary aortas were then collected, and the fatty tissue layers were carefully removed, followed by cutting into cylindrical pieces measuring 2 mm lengthwise to obtain aortic rings. These rings were washed with PBS and further cultured in HiEndoXL^™^ endothelial cell expansion medium in 24-well plates for the experiment. Prior to initiating any treatment condition, these aortic rings were allowed a 12-h incubation period in the complete growth medium. The aortic rings were subjected to an intermittent glucose treatment along with a combination of OICR-9429 (10 μM). After 3 days, the tissue fractions were homogenized, suspended in RIPA lysis buffer, and sonicated. Protein estimation was done by Bradford assay, followed by SDS-PAGE and immunoblotting studies.

### Immunofluorescence Imaging and Analysis

HUVEC were cultured on gelatin (#TC041; HiMedia) coated coverslips up to 70% confluency. After completion of treatment, cells were washed with PBS and subjected to 10 min fixation with 4% ice-cold paraformaldehyde. Cells were incubated with 0.1% Triton X for permeabilization. Cells were blocked with blocking buffer (1% BSA) for 1 h, followed by co-staining with α-SMA and VE-Cadherin. Cells were washed with PBS to remove unbound primary antibody and were subsequently incubated with Alexa fluor 555/488-conjugated antirabbit/mouse secondary antibody (1:4000) for 2 h, followed by incubation with DAPI (#D9542; Sigma-Aldrich) for nuclear staining. Fluorescence images were captured using a Zeiss ApoTome.2 microscope (Carl Zeiss, Jena, Germany), and intensities were measured using ImageJ software. The antibodies used are specified in Supplementary Table S1.

### Inhibitor Treatment and siRNA Silencing in Cultured Endothelial Cells

For predesigned human-specific KMT2D (MLL2) small interfering RNA (Silencer^®^ MLL2 siRNA #AM16708) and control siRNA (SignalSilence^®^ Control siRNA #6568; Cell signaling Technology), MLL2 siRNA was used at a concentration of 40 nM in combination of lipofectamine 2000 (#11668; Invitrogen, Thermo Fisher Scientific). The Scramble siRNA and MLL2-specific siRNA were incubated in Opti-MEM^™^ reduced serum medium (#31985; Gibco) for 4 h and subjected to the abovementioned treatment conditions. Scrambled siRNA was used as a negative control. In combination with MLL2 siRNA inhibition, OICR-9429 (#SML1209; Sigma Aldrich) was used at a concentration of 10 µM. DMSO was used as a vehicle control. Cells were harvested for protein studies.

### Immunoblotting

Media was removed, and cells were briefly washed with sterile 1X PBS. Cells were incubated in RIPA buffer (#R0278; Sigma Aldrich) containing protease inhibitor (#P8340; Sigma Aldrich) for lysis. After 1 h in ice, cells were scraped using a plastic scraper, followed by repeated cycles of sonication (15 kW, two cycles of 15 s each). Cell debris and whole cell lysate were separated by centrifugation (10,000 g for 10 min). Protein concentration was estimated with Bradford assay (Absorbance at 595 nm). Protein samples were processed for SDS polyacrylamide gel electrophoresis with 10–250 KDa prestained protein ladder (#PG-PMT2922; Genetix) as a molecular weight reference. Proteins were transferred onto a nitrocellulose membrane at 15 V, 2.5 A for 30 min. Membrane was blocked with 5% bovine serum albumin (BSA) for 1 h. Membrane was probed overnight at 4°C by incubation with different monoclonal human/rat specific primary antibodies as specified in Supplementary Table S1.

### RNA Isolation

RNA isolation was performed following the manufacturer’s protocol (#15596, TRIzol^™^ Reagent; Life Technologies, Thermo Fisher Scientific). HUVEC were cultured in six-well plates up to 70% confluency and subject to the earlier mentioned treatment regimes. After 72 h, treated cells were incubated in TRIzol^™^ Reagent. Upon harvesting cells, an organic layer was separated using chloroform phase separation, and RNA in the aqueous layer was precipitated using isopropanol. Next, precipitated RNA was washed with 75% ethanol, and RNA pellets were air dried. Finally, RNA pellets were dissolved in sterile nuclease-free water, and quantity and quality were analyzed through a Nano-Drop spectrophotometer (SimpliNano; GE Lifesciences).

### cDNA Synthesis and Quantitative Analysis Through Reverse Transcriptase-Quantitative Polymerase Chain Reaction

To measure the transcript level of different genes, we carried out reverse transcriptase-quantitative polymerase chain reaction (RT-qPCR). Total RNA (1 μg) was taken from the cDNA preparation using iScript^™^ cDNA Synthesis Kit (#1708891; Bio-Rad Laboratories, Hercules, CA, United States). Prior to cDNA synthesis, isolated RNAs were preincubated with DNAse to remove any DNA contamination. Real-time PCR was performed using iTaq^™^ Universal SYBR^®^ Green Supermix (#1725124; Bio-Rad Laboratories) with a total master mix volume of 10 μl. Analysis was carried out by calculating delta-delta Ct. GAPDH was used as the housekeeping gene.

### Chromatin Immunoprecipitation (ChIP) and Subsequent Quantitative PCR

A ChIP assay was performed using an Imprint^®^ Chromatin Immunoprecipitation Kit (#CHP1; Sigma-Aldrich). Cultured HUVEC (80% cell density) were subject to intermittent high-glucose treatment. Treated HUVEC were harvested (1 × 10^6^ cells), washed, and cross-linked with 1% formaldehyde in HiEndoXLTM endothelial cell expansion medium (10 min at room temperature). After washing in PBS, the cell pellet was resuspended in nuclei preparation buffer (200 µl per 10^6^ cells) and kept on ice for 10 min. The nuclear pellet thus obtained was resuspended in shearing buffer (100 µl per 10^6^ cells) supplemented with protease inhibitor cocktail (1 µl per ml of shearing buffer) and further sheared by sonication for 30 s (×40). The sheared chromatin (containing 100–500 bp long sheared genomic DNA) was immunoprecipitated with antibodies directed against H3K4me3 at a concentration of 1:50 (#9751; Cell Signaling Technology). The samples were then washed, reverse cross-linked, and treated with proteinase K to obtain purified DNA fragments. qPCR was performed using primers targeted to amplify regions of human *Jagged1* and *Jagged2* gene promoters.

### Primer Sequences for Transcript and Promoter Primers

Primer sequences to analyze the transcript level of different genes and ChIP-qPCR primer determine promoter level enrichment of H3K4me3 are as mentioned in Supplementary Table S2.

### Statistics

All the values are expressed as the mean ± SD. Statistical significance between groups were determined by one-way ANOVA with a false discovery rate (FDR) method called the two-stage linear step-up procedure of Benjamin, Krieger, and Yekutieli for comparisons of multiple groups and a two-tailed Student’s *t*-test for comparisons between two groups (or a Mann–Whitney *U* test for nonparametric data). In the multiple comparison analysis, a *q*-value representing the FDR of <0.05 (between groups) was considered as a criterion to consider the analysis for inclusion as statistically significant. Statistical analyses were performed using GraphPad Prism software. A *p*-value of less than .05 was considered statistically significant.

## Results

### Intermittent Hyperglycemia Most Potently Induced Acquisition of Mesenchymal Markers in Endothelial Cells

**FIGURE 1:**
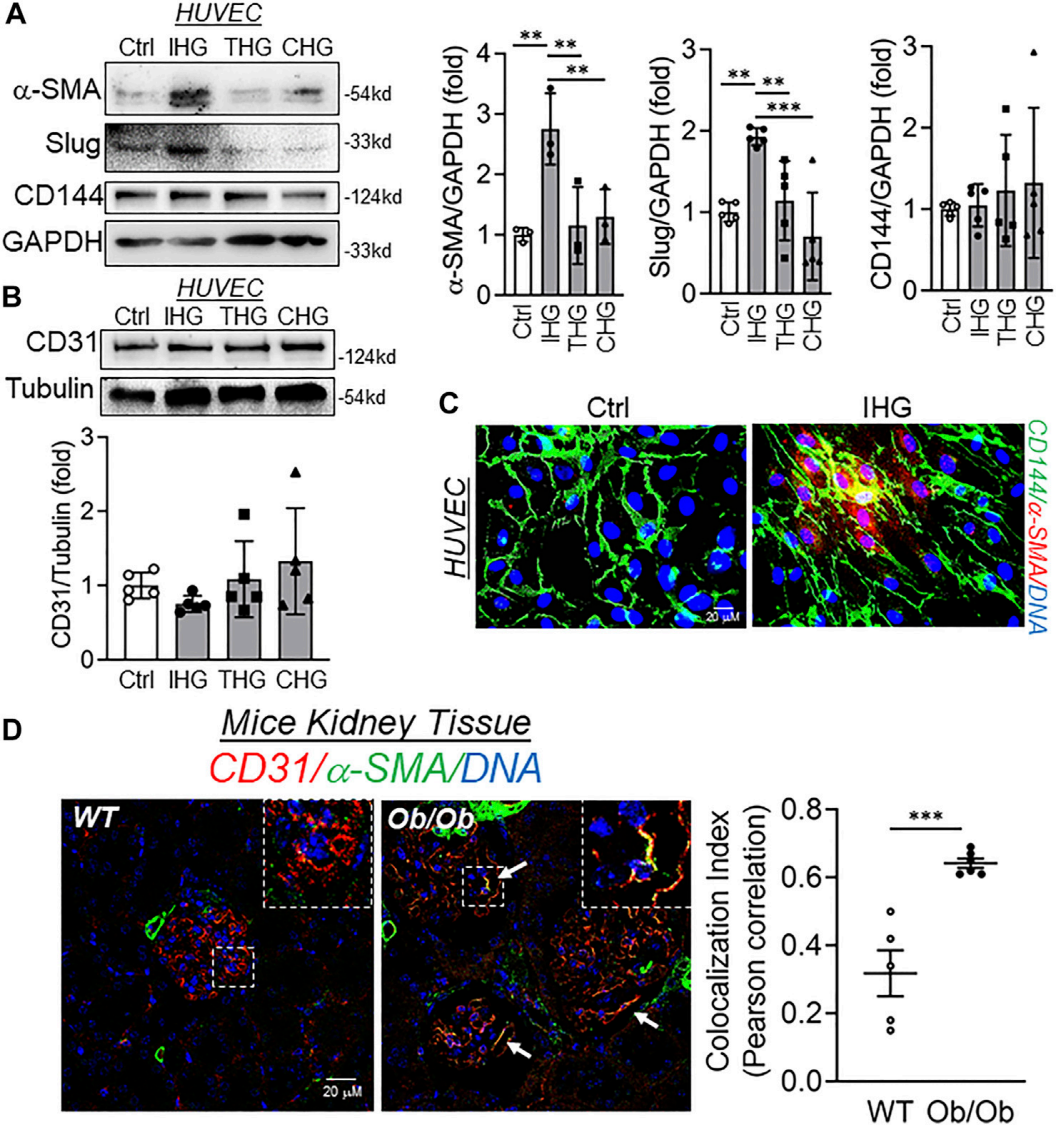
Intermittent high glucose imparts mesenchymal character in endothelial cells both *in vitro* and *in vivo*. **(A**,**B)** Immunoblot analysis of HUVEC lysates collected from cells treated with differential high-glucose treatment conditions and probed for α-SMA (**A**, *n* = 3), Slug (**A**, *n* = 5), CD144 (**A**, *n* = 5), and CD31 (**B**, *n* = 5) along with their respective densitometry quantification analysis. **(C)** Immunofluorescence analysis and costaining of HUVEC exposed to intermittent high glucose for α-SMA and CD144. DAPI staining to visualize the nucleus is shown in blue (*n* = 3). **(D)** Immunohistochemistry of tissue sections from BTBR WT and BTBR Ob/Ob mice stained for α-SMA and CD31 (*n* = 3). DAPI staining is shown in blue. Images were acquired with Zeiss LSM 780 Microscope. White arrowheads indicate endothelial cells expressing both CD31 and α-SMA. Degree of colocalization was analyzed using the Coloc2 plugin of ImageJ and Pearson correlation coefficient values were plotted to indicate the level of colocalization between CD31 and α-SMA. Values represent the mean ± SD. **p < .01, and ***p < .001, either by unpaired *t*-test for two groups or the two-stage linear step-up procedure of Benjamin, Krieger, and Yekutieli test for comparisons of multiple groups.

In a patient with type 2 diabetes, oscillating blood glucose level is apparent and shown to have more deleterious effects than constant high glucose on endothelial function and oxidative stress, two key factors associated with hyperglycemia-dependent cardiovascular complications (Quagliaro et al., 2003; Ceriello et al., 2008). Furthermore, our recent work indicates that pulses of high-glucose shock (we termed this intermittent high glucose) promotes an inflammatory switch of EC through epigenetically regulating *KLF2* and *KLF4* genes (Thakar et al., 2021). Because oscillating glucose levels causes potent endothelial dysfunction, we, therefore, undertook experiments to evaluate the effect of different high-glucose treatment conditions, such as intermittent high glucose (12 h of 25 mM glucose followed by 12 h of 5.5 mM glucose in consecutive three cycles totaling 72 h time), transient high glucose (24 h of 25 mM glucose followed by 48 h of 5.5 mM glucose) and constant high glucose (72 h of 25 mM high glucose) on acquistion of mesenchymal character. Through detection of mesenchymal markers, we established that intermittent high glucose maximally induced some of the mesenchymal-like biochemical character in EC as confirmed through the expression of α-SMA and Slug (Figure 1A). Interestingly, for the given time point, we were unable to detect any reduction in the expression of CD144 (Figure 1A) and CD31 (Figure 1B) and their expression remained unaltered. We also measured the level of other EndMT-associated transcripts and/or proteins, including N-Cadherine, Vimentin, Snail, Calponin, Versican, and FSP1. In so doing, we did not detect significant alteration in transcript and/or protein level expression of N-Cadherine, Vimentin, Calponin, Versican, and FSP1 (Supplementary Figures S1A,D–F) while elevating Slug transcript level (Supplementary Figure S1C). Surprisingly, unlike Slug, we observed a significant reduction in the level of Snail protein when HUVEC were challenged with intermittent high glucose (Supplementary Figure S1B). Because, unlike the EndMT phenotype of EC, we did not detect a complete change in the biochemical character of the intermittent high-glucose-challenged EC, we, therefore, named such phenomenon as “EC exhibiting partial mesenchymal character.”

To exclude the possibility of intermittent hyperglycemia-dependent induction of partial mesenchymal character being a simple bystander due to the osmolality effect, we next measured the level of CD31, CD144, α-SMA, and Slug in EC exposed to intermittent D-Mannitol. We fixed D-Mannitol concentration such that it achieves comparable osmolality in a cell system as similar to high-glucose treatment. Through such experiments, we detected no change in protein level expression of CD31, CD144, α-SMA, and Slug in HUVEC exposed to D-Mannitol (Supplementary Figure S2A).

Because we did not detect any alteration in CD144 and CD31 while the expression of α-SMA and Slug were found to be elevated, we wondered whether such phenomenon is due to the acquisition of the mesenchymal character of EC prior to losing their endothelial character. To confirm whether EC is undergoing mesenchymal transition prior to losing endothelial biomarkers, we then performed dual immunofluorescence staining to detect CD144 and α-SMA. In so doing, we detected that intermittent high glucose caused expression of α-SMA in HUVEC, which also expressed CD144 in comparison to normal glucose-incubated cells (Figure 1C). To ascertain these *in vitro* findings in an *in vivo* model of hyperglycemia, we further used WT and Ob/Ob mice. These mice exhibited heightened blood glucose levels (Supplementary Figure S3A) and body weight (Supplementary Figure S3B). Interestingly, Ob/Ob mice displayed significant loss of albumin through urine in comparison to their age- and sex-matched WT mice (Supplementary Figures S3C,D). In addition, we also found a significant accumulation of extracellular matrix in the glomeruli of Ob/Ob mice (Supplementary Figure S3E). We further performed CD31 and α-SMA co-staining in kidney tissues of WT and Ob/Ob mice and imaged the glomeruli of kidney tissue sections. Through such imaging, we further confirmed a considerable number of glomerular EC concomitantly expressing both CD31 and α-SMA in Ob/Ob mice (Figure 1D and Supplementary Figure S4A). Furthermore, colocalization analysis revealed significant colocalization of CD31 and α-SMA in endothelial cells in the glomerular region of kidney tissues from Ob/Ob mice (Figure 1D, bar graph and Supplementary Figures S4B,C).

### Intermittent Hyperglycemia Exposure Selectively Elevated MLL2 and WDR82 in Endothelial Cells to Facilitate the Catalysis of H3K4me3

**FIGURE 2:**
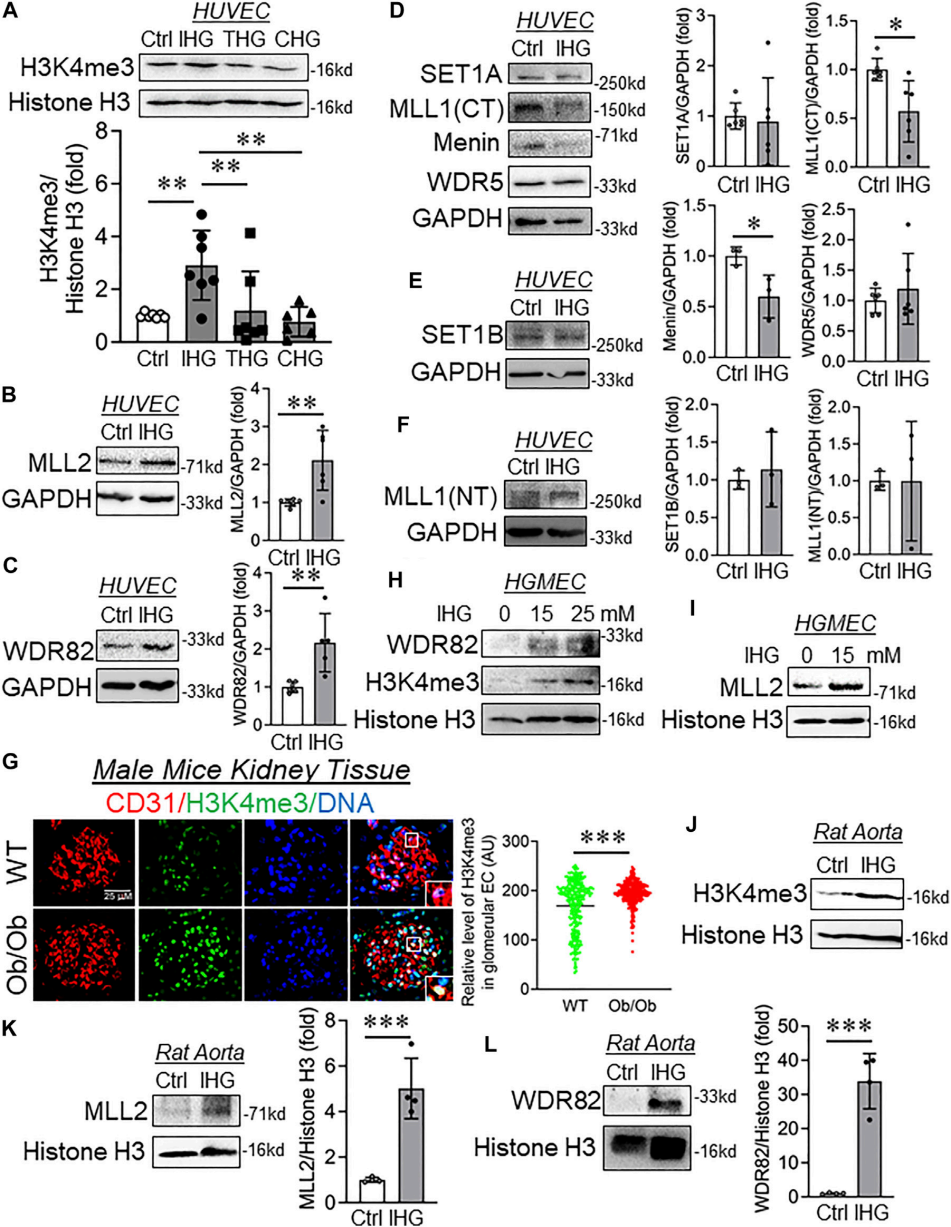
Intermittent hyperglycemia caused increased levels of H3K4me3 through upregulation of MLL2 and WDR82. **(A**–**F)** Immunoblot analysis of HUVEC lysates collected from cells treated with differential/intermittent high-glucose treatment conditions and probed for H3K4me3 (**A**, *n* = 7), MLL2 (**B**, *n* = 6), WDR82 (**C**, *n* = 5), SET1A (**D**, *n* = 5), MLL1 (CT) (**D**, *n* = 6), Menin (**D**, *n* = 3), WDR5 (**D**, *n* = 6), SET1B (**E**, *n* = 3), and MLL1 (NT) (**F**, *n* = 3). **(G)** Immunohistochemistry of tissue sections from control and Ob/Ob mice stained for H3K4me3 and CD31 (*n* = 6). Fluorescence intensity AU values per individual cell nucleus are indicated together with the mean. Total number of nuclei, *n* ≥ 150. **(H**,**I)** Immunoblotting for H3K4me3 **(H)**, WDR82 **(H),** and MLL2 **(I)** in cultured HUVEC exposed to intermittent high glucose with a high-glucose concentration of 15 and 25 mM (*n* = 3). **(J**–**L)** Immunoblot analysis of rat aorta tissue lysates collected from cells treated with intermittent high-glucose treatment conditions and probed for H3K4me3 **(J)**, MLL2 **(K)** and WDR82 **(L)** along with their respective densitometry quantification. Values represent the mean ± SD. *p < .05, **p < .01, and ***p < .001, either by unpaired *t*-test for two groups or the two-stage linear step-up procedure of Benjamin, Krieger, and Yekutieli test for comparisons of multiple groups.

Once we established that intermittent hyperglycemia most effectively caused expression of some the mesenchymal genes, we next set out to explore the possible underlying mechanisms. Alteration in epigenetic marks is prevalent in EC undergoing mesenchymal transition while hyperglycemia independently regulates multiple epigenetic mechanisms. A recent study by Glaser and coworkers describes site-specific reduction of repressive H3K9me3 marks at promoters of mesenchymal genes through promoter-level enrichment of the demethylase JMJD2B (Glaser et al., 2020). However, whether hyperglycemia alters epigenetic pathways to cause a mesenchymal phenotype in EC is yet to be established. To address this, we explored the methylation of histone 3 lysine 4 in cells and tissues upon hyperglycemia. Intermittent hyperglycemia treatment significantly elevated the level of activating the H3K4me3 mark in HUVEC (Figure 2A). To eliminate the possibility of an osmolality effect, we next measured the H3K4me3 level in EC incubated with intermittent D-Mannitol. By doing so, we detected no change in the H3K4me3 level in HUVEC exposed to D-Mannitol (Supplementary Figure S2B).

To identify the cause of such an increase in H3K4me3, we measured the level of the SET1/COMPASS family of proteins that are responsible for catalysis of H3K4me3. We detected a significant increase in MLL2 (Figure 2B) and WDR82 (Figure 2C) in HUVEC exposed to intermittent hyperglycemia, and such treatment did not alter the protein level expression of SET1A (Figure 2D), WDR5 (Figure 2D), SET1B (Figure 2E), and MLL1(NT) (Figure 2F). Surprisingly, despite the increase in cellular H3K4me3 level, MLL1(CT) and Menin protein significantly reduced upon intermittent hyperglycemia challenge (Figure 2D). To validate such findings *in vivo* and because we know hyperglycemia causes renal microvascular dysfunction, we performed dual staining of H3K4me3 with CD31 in Ob/Ob mice kidney tissues. Through such analysis, we detected an endothelial-specific heightened H3K4me3 level in kidney glomeruli (Figure 2G). Furthermore, *in vitro* analysis of differential intermittent high-glucose-challenged human glomerular EC (HGMEC) showed a heightened H3K4me3 level (Figure 2H) associated with an increase in MLL2 (Figure 2I) and WDR28 levels (Figure 2H). We also confirmed such an elevation in H3K4me3 (Figure 2J), MLL2 (Figure 2K), and WDR82 (Figure 2L) in the rat aorta exposed to the intermittent high-glucose treatment condition *ex vivo*.

We further examined the level of H3K4-specific lysine-specific demethylase 1A (LSD1), which preferentially caused demethylation of H3K4me1 and H3K4me2. Interestingly, we observed no alteration in the LSD1 protein level in HUVEC exposed to the intermittent high-glucose treatment condition (Supplementary Figure S5A). We also detected H3K4me1 and H3K4me2 in HUVEC challenged with intermittent high glucose and observed no detectable changes in H3K4me1 (Supplementary Figure S5B) and H3K4me2 (Supplementary Figure S5C).

### H3K4me3 Dependent Transcriptional Switch of Jagged Ligands Caused Activation of Notch Signaling in Endothelial Cells Exposed to Intermittent Hyperglycemia

**FIGURE 3:**
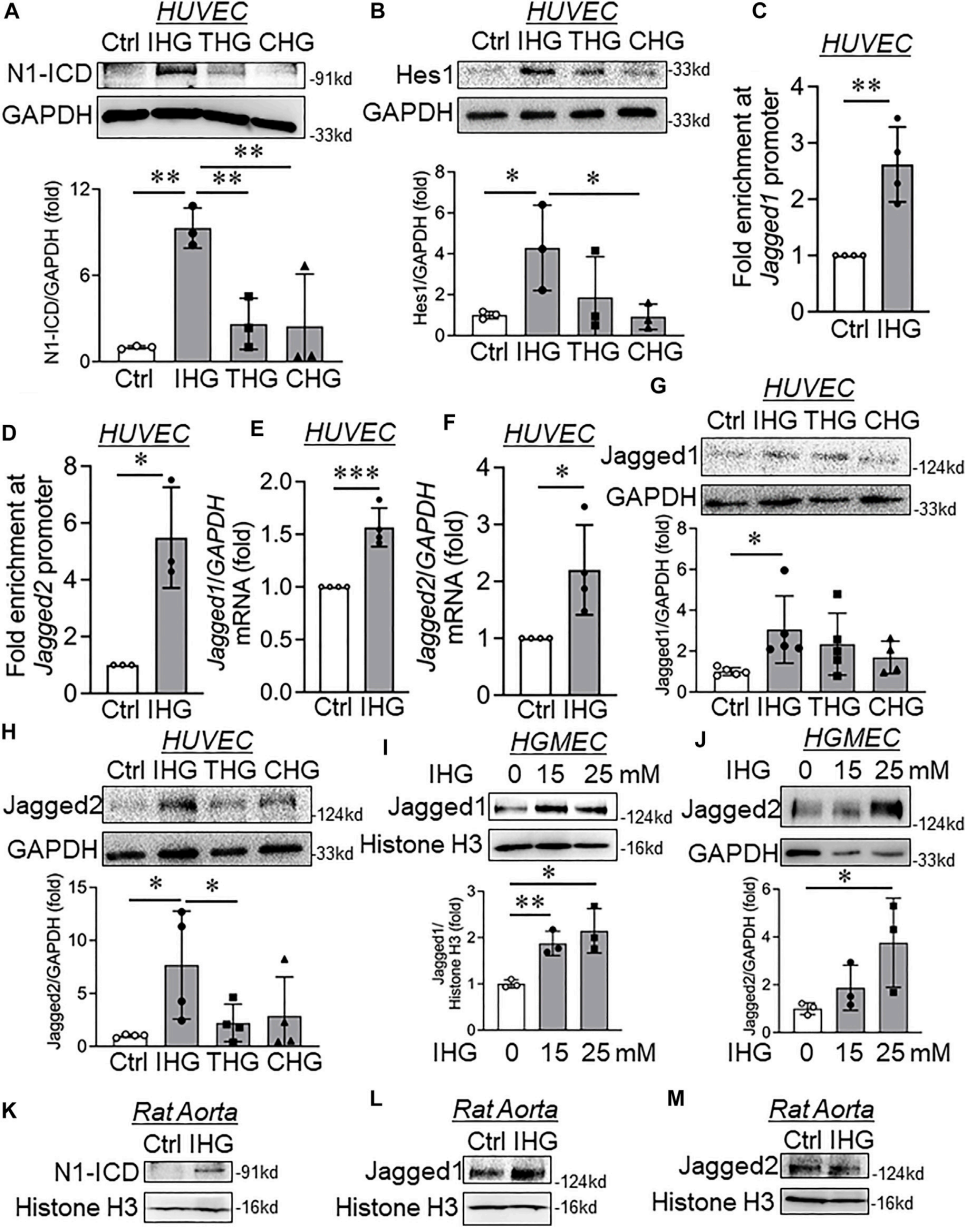
Elevated H3K4me3 upon intermittent high-glucose exposure enriched in Jagged1 and Jagged2 promoter, thereby causing their expression and Notch activation in endothelial cells. **(A**,**B)** Immunoblot analysis of endothelial cell lysates collected from cells treated with intermittent, transient, and constant high glucose and probed for N1-ICD (**A**, *n* = 3) and Hes1 (**B**, *n* = 3). **(C**,**D)** ChIP-qPCR analysis of H3K4me3 enrichment on *Jagged1* (**C**, *n* = 4) and *Jagged2* (**D**, *n* = 3) gene promoters in HUVEC exposed to intermittent hyperglycemia. **(E**,**F)** Transcript-level expression of *Jagged1* (**E**, *n* = 4) and *Jagged2* (**F**, *n* = 4) measured through RT-qPCR technique in HUVEC challenged with an intermittent high-glucose condition. **(G**,**H)** Immunoblot analysis of HUVEC lysates collected from cells treated with differential high-glucose treatment conditions and probed for Jagged1 (**G**, *n* = 5) and Jagged2 (**H**, *n* = 4). **(I**,**J)** Human glomerular endothelial cells (HGMEC) were treated with intermittent high-glucose treatment conditions and immunoblotted for Jagged1 (**I**, *n* = 3) and Jagged2 (**G**, *n* = 3). **(K**–**M)** Immunoblot analysis of rat aorta tissue lysates collected from cells treated with intermittent high-glucose treatment conditions and probed for N1-ICD (**K**, *n* = 3) Jagged1 (**L**, *n* = 3), Jagged2 (**M**, *n* = 3). Values represent the mean ± SD. *p < .05, **p < .01, and ***p < .001, either by unpaired *t*-test for two groups or the two-stage linear step-up procedure of Benjamin, Krieger, and Yekutieli test for comparisons of multiple groups.

Because we detected an increase in the expression of a few of the mesenchyme-associated genes, including α-SMA and Slug, we, therefore, decided to evaluate the promoter-level enrichment of H3K4me3 on α-SMA and Slug genes in EC challenged with intermittent hyperglycemia. ChIP-qPCR analysis of *α-SMA* and *Slug* gene promoters indicated no alteration of H3K4me3 enrichment on these promoters (Supplementary Figures S6A,B). Activation of Notch signaling is well-described as a driver of mesenchymal transition of EC (Chang et al., 2011). Therefore, we measured the level of active Notch signaling in EC exposed to intermittent hyperglycemia. Intermittent hyperglycemia-challenged EC exhibited Notch activation as detected through an increase in Notch1-intracellular domain (N1-ICD) level (Figure 3A) and heightened expression level of Notch downstream target gene Hes1 (Figure 3B). Next, we questioned whether such activation of Notch signaling upon intermittent hyperglycemia exposure could be associated with control of Notch ligands through promoter-level regulation by H3K4me3. Through ChIP-qPCR analysis, we indeed confirm that H3K4me3 was significantly enriched in the proximal promoter region of both *Jagged1* (Figure 3C) and *Jagged2* (Figure 3D) gene in EC challenged with intermittent hyperglycemia. Furthermore, concurrent increase in *Jagged1* and *Jagged2* transcripts (Figures 3E,F) and protein (Figures 3G,H) levels were detected, respectively, in intermittent high-glucose-treated EC. In addition, we measured the protein level expression of membrane-associated metalloproteinases, ADAM9, ADAM10, and ADAM17, which cause ligand-independent activation of Notch signaling. Through such analysis, we did not observe any alteration in ADAM9 (Supplementary Figure S6C), ADAM10 (Supplementary Figure S6D), and ADAM17 (Supplementary Figure S6E) metalloproteinases. We also detected the expression of other Notch ligands, such as Dll1 and Dll4. Although we are unable to detect Dll1 with the specified antibody (Supplementary Figure S6F), interestingly, intermittent high-glucose-treatment caused a significant reduction in another Notch ligand Dll4 (Supplementary Figure S6H), which is previously shown to have a more protective role in maintaining normal physiological functioning of EC (Benedito et al., 2009). Furthermore, we did not detect changes in many other Notch downstream genes, such as Cyclin D3 (Supplementary Figure S6F) and cMyc (Supplementary Figure S6G), indicating induction of Hes1 expression possibly as the primary target of Notch1 activation in current settings.

We then detected the changes in Notch signaling–associated genes in HUVEC that are exposed to intermittent D-Mannitol treatment condition as specified earlier. We could detect either unchanged or statistically insignificant changes in Jagged1 (Supplementary Figure S2C), Jagged2 (Supplementary Figure S2D), N1-ICD, and Hes1 (Supplementary Figure S2C) protein levels. We further confirm the increase in Jagged1 (Figure 3J) and Jagged2 (Figure 3J) protein levels in HGMEC exposed to intermittent high-glucose conditions. To provide evidence that such changes also occur at the tissue level, we detected active Notch1 product N1-ICD and its associated ligands Jagged1 and Jagged2 in rat aorta tissues that were challenged with intermittent hyperglycemia *ex vivo*. Parallel to *in vitro* observation, we detected significant increase in N1-ICD (Figure 3K) and Jagged1 (Figure 3L) level in groups in which rat aortic tissues were incubated in intermittent high-glucose treatment conditions. Interestingly, we could not detect any alteration in Jagged2 protein level (Figure 3M) in intermittent high-glucose treated rat aortic tissues.

### Catalytic Inhibition of MLL Protein Through Small Molecule or siRNA Mediated Knockdown of MLL2 Reversed Intermittent High Glucose-dependent Increase in Notch Activation *In Vitro*

**FIGURE 4:**
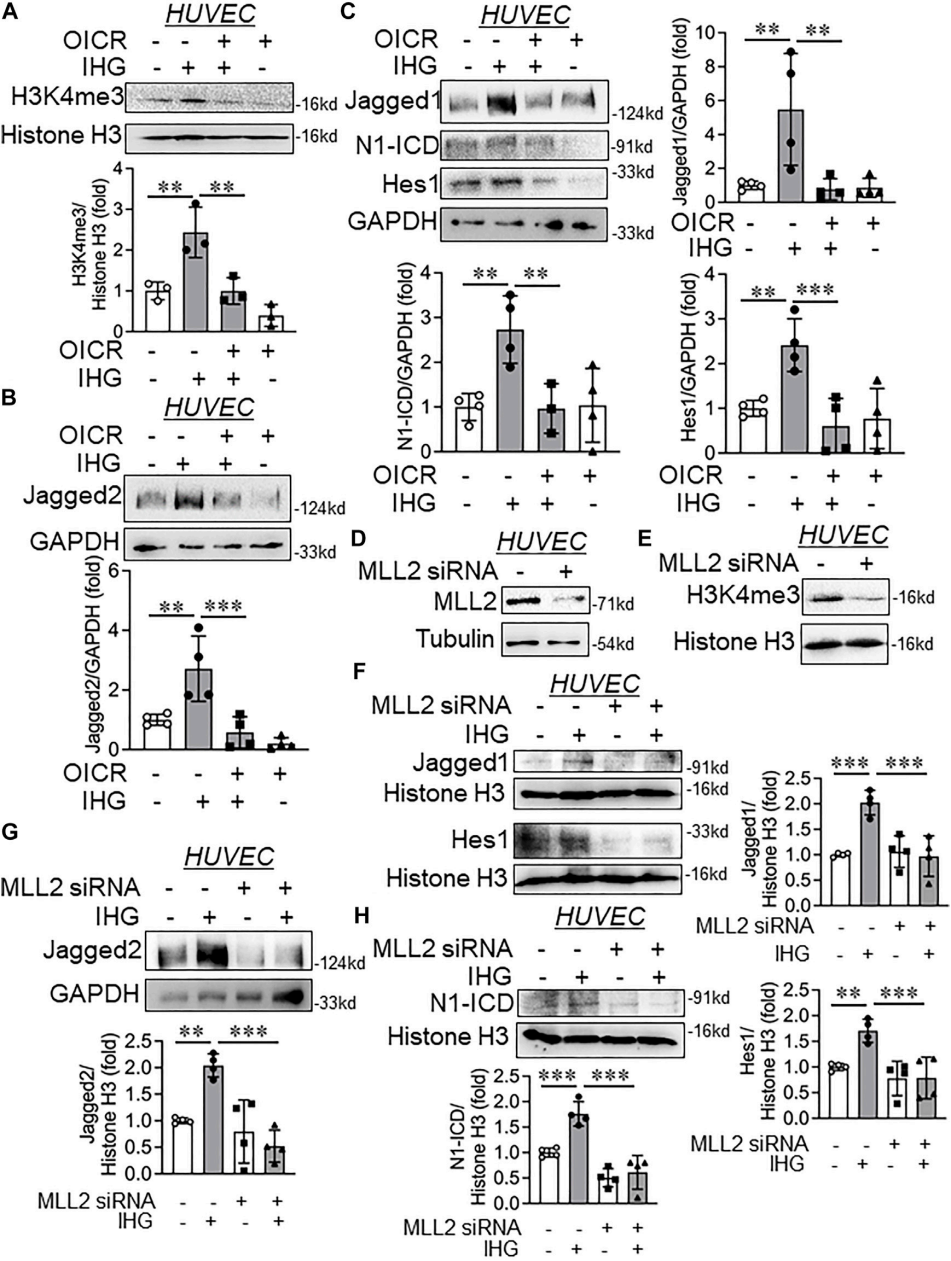
Catalytic inhibition or siRNA-mediated knockdown of MLL blocked intermittent high-glucose-dependent increase in Jagged1 and Jagged2 expression and further attenuating Notch activation. **(A**–**D)** Immunoblot analysis of HUVEC lysates collected from cells treated with intermittent high glucose in combination with OICR-9429 and probed for H3K4me3 (**A**, *n* = 3), Jagged2 (**B**, *n* = 4), Jagged1 (**C**, *n* = 4), N1-ICD (**C**, *n* = 4), Hes1 (**C**, *n* = 4) along with densitometry quantification. **(D**,**E)** HUVEC were transfected with MLL2 siRNA by following lipofectamine 2000 protocol followed by immunoblot to detect MLL2 (**D**, *n* = 3) and H3K4me3 (**E**, *n* = 3). **(F**–**H)** Immunoblot analysis of HUVEC lysates collected from cells transfected with MLL2 specific siRNA followed by challenging with intermittent high glucose and probed for Jagged1 (**F**, *n* = 4), Jagged2 (**G**, *n* = 4), N1-ICD (**H**, *n* = 4), Hes1 (**F**, *n* = 4) along with densitometry quantification of the blots. Values represent the mean ± SD. **p < .01, and ***p < .001, by the two-stage linear step-up procedure of Benjamin, Krieger, and Yekutieli test.

As we established that H3K4me3-dependent transcriptional activation of Notch ligands Jagged1 and Jagged2 caused their de-repression followed by Notch signaling activation, we wondered whether inhibiting H3K4me3 catalysis through MLL proteins could abrogate intermittent high-glucose-driven Notch ligand expression and activation of Notch signaling. We took advantage of a well-established MLL inhibitor named OICR-9429 (Grebien et al., 2015) and studied its effect on intermittent high glucose-dependent Notch signaling in EC. Administration of OICR-9429 blocked both basal and intermittent high glucose-dependent increase in H3K4me3 level (Figure 4A). Concomitantly, EC treated with OICR-9429 and exposed to intermittent high glucose displayed lower levels of Jagged1 (Figure 4C), Jagged2 (Figure 4B), N1-ICD (Figure 4C), and Hes1 (Figure 4C) proteins compared with only intermittent high-glucose-challenged EC, indicating inhibition of Notch signaling activation in intermittent hyperglycemia-challenged HUVEC. Because, in our previous work, we established that intermittent high-glucose exposure to EC caused significant reduction in KLF2 and KLF4 level (Thakar et al., 2021) and such reduction in KLF2 and KLF4 were previously shown to be associated with EndMT (Moonen et al., 2015), we, therefore, measured the levels of KLF2 and KLF4 in EC exposed to intermittent hyperglycemia and OICR. Such experiment revealed that inhibition of H3K4me3 catalysis in intermittent hyperglycemia-challenged EC normalized KLF2 level (Supplementary Figure S7A) without reversing the level of KLF4 (Supplementary Figure S7B).

We next used a prevalidated human MLL2 specific siRNA and determined whether such targeted knockdown of MLL2 can attenuate intermittent high-glucose dependent de-repression of Jagged1 and Jagged2 to further diminish intermittent high-glucose-driven Notch activation. We detected a significant reduction in MLL2 protein level (Figure 4D) accompanied with reduction in H3K4me3 levels (Figure 4E) upon MLL2 knockdown in HUVEC. Furthermore, MLL2 knockdown diminished intermittent hyperglycemia-mediated increase in Jagged1 (Figure 4F), Jagged2 (Figure 4G), N1-ICD (Figure 4H), and Hes1 (Figure 4F) protein levels in HUVEC. In parallel to the OICR treated groups, MLL2 knockdown normalized the level of KLF2 in intermittent high-glucose-challenged EC (Supplementary Figure S7C).

### Intermittent Hyperglycemia-Mediated Increase in Notch Activation Was Attenuated by MLL Catalytic Inhibition *ex vivo*

**FIGURE 5:**
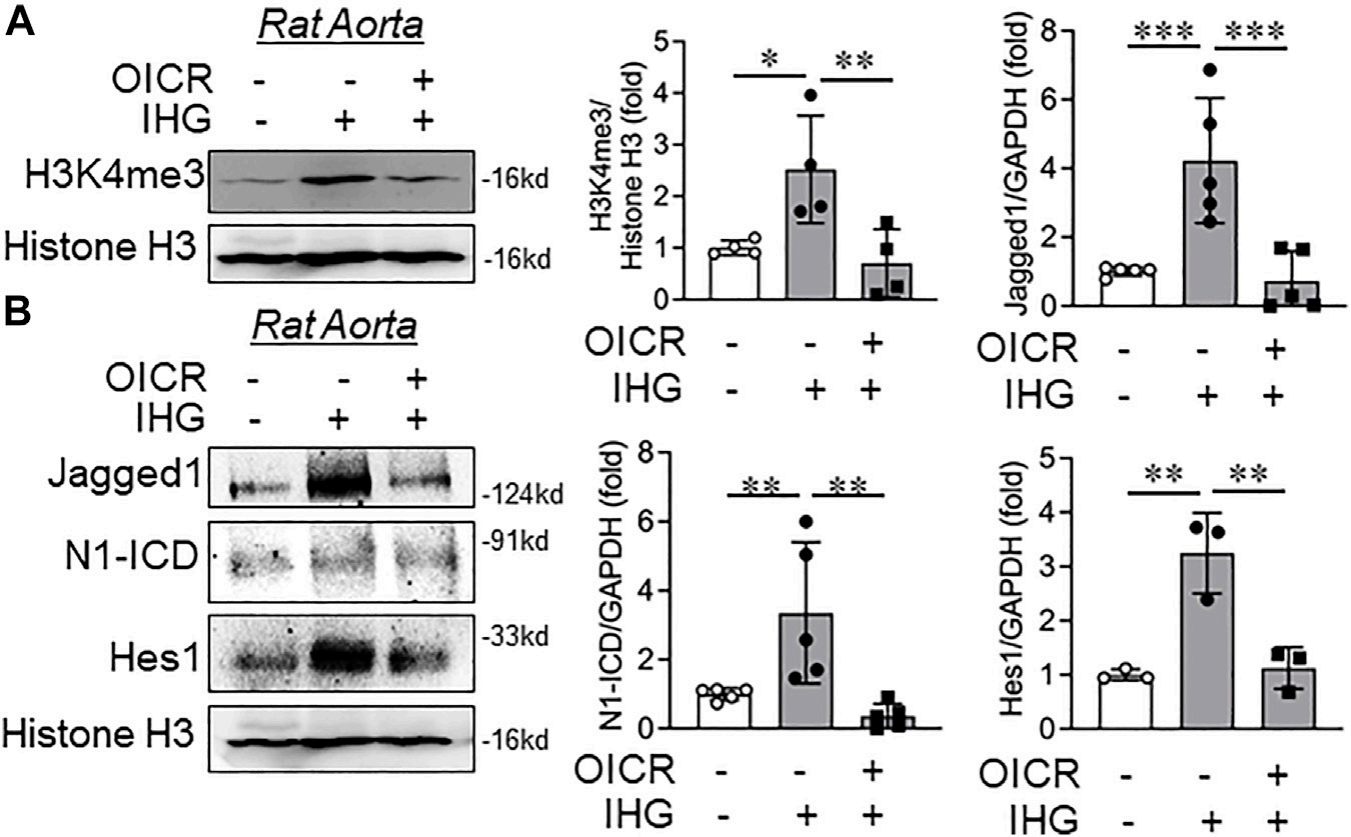
Catalytic inhibition of MLL blocked intermittent high-glucose-dependent increase in Notch *ex vivo* in rat aorta. **(A**,**B)** Immunoblot analysis of tissue lysates collected from rat aorta treated with intermittent high glucose in combination with OICR-9429 and probed for H3K4me3 (**A**, *n* = 4), Jagged1 (**B**, *n* = 5), N1-ICD (**B**, *n* = 5), and Hes1 (**B**, *n* = 3) along with densitometry quantification. Values represent the mean ± SD. *p < .05, **p < .01, and ***p < .001, by the two-stage linear step-up procedure of Benjamin, Krieger, and Yekutielitest.

Once we confirmed the reversal of expression for specified Notch-associated genes upon MLL pharmacological and molecular inhibition in intermittent hyperglycemia-challenged EC *in vitro*, we then studied the effect of MLL inhibition on H3K4me3 catalysis and Notch activation in an *ex vivo* model of rat aortic rings. Parallel exposure of intermittent hyperglycemia and OICR-9429 blocked the high-glucose-dependent increase in H3K4me3 levels in tissue lysates of rat aortic rings (Figure 5A). Upon OICR-9429 administration, intermittent high-glucose treatment failed to elevate the level of Notch ligands Jagged1 (Figure 5B) and further could not potentiate Notch receptor activation and downstream signaling as detected through N1-ICD and Hes1, respectively (Figure 5B).

### Inactivation of MLL Through Small Molecule or siRNA Knockdown Revoked Intermittent High-Glucose-Driven Partial Mesenchymal Character of Endothelial Cells

**FIGURE 6:**
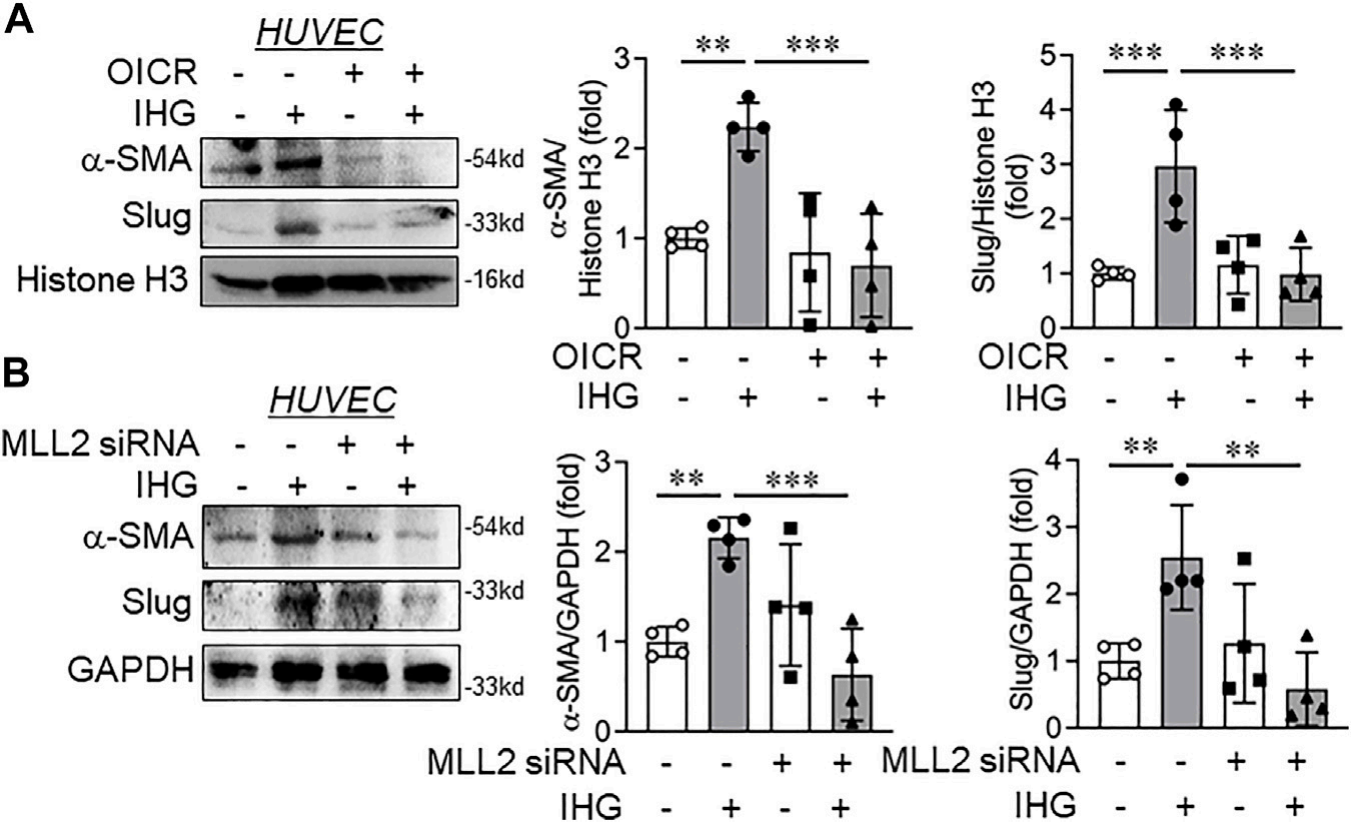
Catalytic inhibition or siRNA-mediated knockdown of MLL blocked intermittent high-glucose-dependent mesenchymal switch of endothelial cells. **(A)** HUVEC were exposed to intermittent high-glucose treatment condition in combination with OICR-9429 and incubated for a total of 5 days followed by immunoblot analysis by probing for α-SMA and Slug along with densitometry quantification of the blots (*n* = 4). **(B)** HUVEC were transfected with MLL2 specific siRNA followed by challenging with intermittent high glucose and incubation for a total of 5 days. Resultant cell lysate was immunoblotted and probed for α-SMA and Slug along with densitometry quantification of the blots (*n* = 4). Values represent the mean ± SD. **p < .01, and ***p < .001, by the two-stage linear step-up procedure of Benjamin, Krieger, and Yekutieli test.

Upon confirming Notch inactivation through MLL inhibition, we next set out to determine whether MLL inactivation could override the intermittent high-glucose effect on acquisition of the partial mesenchymal character of EC. Because we observed significant expression of α-SMA and Slug in intermittent high-glucose-subjected HUVEC, we, therefore, measured these two key mesenchymal markers in EC that are exposed to intermittent high glucose and either preconditioned with OICR-9429 or MLL2-specific siRNA. Catalytic inhibition of MLL through OICR-9429 administration to EC attenuated hyperglycemia dependent induction of mesenchymal genes α-SMA and Slug (Figure 6A) in EC without altering the level of endothelial surface markers CD31 and CD144 (data not shown).

We next analyzed the expression of these mesenchymal genes in EC in which MLL2 was knocked down using MLL2-specific siRNA and were challenged to an intermittent high-glucose condition. In parallel to OICR-9429 treatment, α-SMA and Slug expression levels diminished in intermittent hyperglycemia-exposed EC where MLL2 was knocked down in comparison to only intermittent high-glucose-treated EC (Figure 6B). However, no changes in CD31 and CD144 were detected between any of these treatment groups (data not shown). In addition, no changes in N-cadherin and vimentin were detected in EC that are either exposed to intermittent high glucose or challenged with intermittent high glucose in the presence of OICR or MLL2 siRNA (Supplementary Figures 7D,E)

## Discussion

**FIGURE 7:**
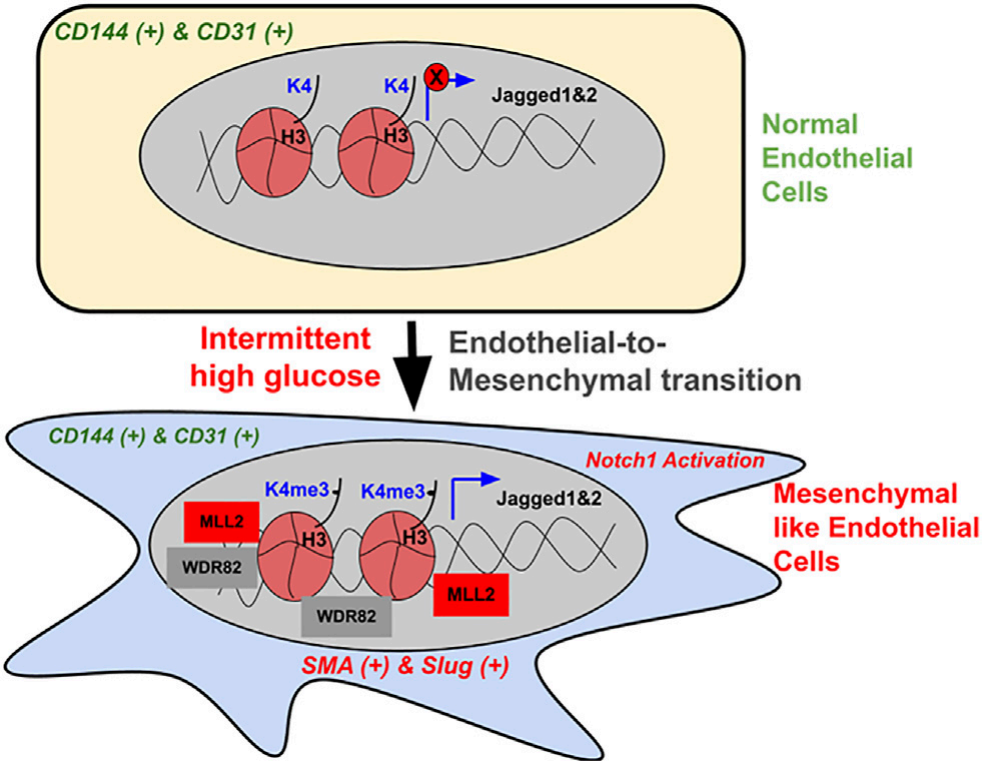
Schematic illustration of the intermittent high-glucose-dependent induction of H3K4me3 promoting Notch signaling-dependent acquisition of mesenchymal character of endothelial cells. In normal endothelial cells, H3K4me3 levels remain to be minimal and the expression of Notch-associated ligands Jagged1 and Jagged2, including the activation of Notch pathway is very limited, thereby maintaining endothelial phenotype. Upon intermittent high-glucose challenge, endothelial cells exhibit a heightened level of H3K4me3 contributed by an increase in SET1/COMPASS proteins MLL2 and WDR82. Elevated H3K4me3 consequently enriches on the promoter region of Notch ligands Jagged1 and Jagged2, thereby causing their expression, which in turn causes Notch activation. Such hyperactivation of Notch signaling supports the acquisition of mesenchymal character in endothelial cells. Interplaying with MLL through small molecule inhibitors or by siRNA-dependent knockdown caused reversal of intermittent high-glucose dependent Notch activation and concurrent inhibition of acquiring mesenchymal character by endothelial cells.

Alterations in endothelial biochemical and functional characteristics are a hallmark of many human diseases, including diabetes-dependent secondary complications (Hadi and Al Suwaidi, 2007) (Kolluru et al., 2012). Reprogramming the fate of endothelial lineage through epigenetic mechanisms is emerging as one of the most important molecular pathways driving cardiovascular diseases (Zeisberg et al., 2007; Evrard et al., 2016). For example, endothelial identity and function are critically controlled by the histone demethylase JMJD2B by regulating the level of H3K9me3, which is induced by EndMT-promoting, proinflammatory, and hypoxic conditions and supports the acquiring of a mesenchymal phenotype (Glaser et al., 2020). However, we are still far from understanding the complex epigenetic pathways that interplay with signaling cascades governing cell fate and their trans-differentiation during human diseases, especially in diabetic conditions. In the present study, we identified that exposure of EC to intermittent high glucose, which is prevalent in people with type-2 diabetes, caused a phenotypic switch of these cells to acquire partial mesenchymal character without abandoning the endothelial character. Such cells exhibited a heightened level of Notch activation through increased expression of Notch ligands Jagged1 and Jagged2 genes. This was, in part, mediated by an abrupt elevation in cellular H3K4me3 levels supported by an increase in specific SET1/COMPASS family of proteins MLL2 and WDR82. Augmented H3K4me3 enriched in the proximal promoter region of Jagged1 and Jagged2 genes further causing their de-repression and Notch activation. Interplaying with MLL through pharmacological or molecular inhibition approach abrogated intermittent high-glucose-mediated increase in H3K4me3 level and Notch activation and blocked the partial mesenchymal phenotype of EC (Figure 7).

EndMT is a hallmark of many cardiovascular diseases (Alvandi and Bischoff, 2021). It became apparent that EndMT contributes in different stages of development, and mounting evidence confirms its role in driving cardiovascular diseases, including atherosclerosis, pulmonary hypertension, and valvular disease (Hulshoff et al., 2018). For instance, EndMT was apparent during morphogenesis of atrioventricular valvuloseptal in different stages of development (Eisenberg and Markwald, 1995). The work of Paruchuri et al., 2006 for the first time described transition of clonal endothelial-like cells isolated from human pulmonary valves to a mesenchymal phenotype, analogous to EMT, specifically in response to TGFβ2. They also reported TGFβ2-induced expression of EMT markers and increased cellular migration and invasion capacity and indicated such EC inability to form tube-like structures and to adhere leukocytes in response to inflammatory signals (Paruchuri et al., 2006). In a disease setting, such as diabetes, EndMT is a key phenomenon responsible for early development of diabetic nephropathy. Reports indicate that advanced glycation end products (AGEs), a by-product of hyperglycemia-induced EndMT through receptor for AGE (RAGE)-Smad3 cross-talk and inhibiting such pathway retarded the early development of renal fibrosis in STZ-induced diabetic mice (Li et al., 2010). In addition, EndMT was indicated as pathogenetic significance in diabetic cardiomyopathy and epigenetic mechanism involving miR-200b was indicated as the key driver (Feng et al., 2016). In parallel to these findings, our study strongly indicates acquisition of partial mesenchymal-like character of EC when exposed to hyperglycemia condition *in vitro* representing oscillating blood glucose level as apparent in type-2 diabetic individuals. In addition, in type-2 diabetic rodent model *in vivo*, EC from kidney glomeruli exhibited co-expression of both mesenchymal (α-SMA and Slug) and endothelial-specific (CD31 and CD144) biochemical markers. Interestingly, in the present setting as reported earlier (Paruchuri et al., 2006), EC displayed expression of both endothelial-specific as well as mesenchymal-specific gene expression representing a state in which EC acquire mesenchymal character much prior to losing their original endothelial phenotype.

Hyperglycemia associated with diabetes causes rapid and dynamic epigenetic alterations, including changes in histone methylation in different cell types (Li et al., 2010; Feng et al., 2016). Indeed, transient hyperglycemia exposure to EC causes a long-lasting activation of p65 through Set7 recruitment to p65 promoter and caused an activating chromatin by elevating the level of H3K4me1. In another study, the author reports a sustained upregulation of the NFκB-p65 gene due to hyperglycemia-mediated elevation in H3K4me1 but not H3K4me2 or H3K4me3 level. They also report that high glucose caused suppression of H3K9me2 and H3K9me3 methylation on the p65 promoter, thereby representing a p65 gene promoter that was poised to be activated, thus leading to sustained expression of these genes (Brasacchio et al., 2009). In this present study, we describe that an oscillating high-glucose exposure to EC causes an elevation in H3K4me3 level without altering the level of H3K4me1 and H3K4me2 and this increase was driven by an augmented expression of MLL2 and WDR82, two of the important proteins of SET1/COMPASS complex, supporting H3K4me3 catalysis. Previous work from our lab also shows that oscillating blood glucose levels induced H3K27me3 levels in EC by supporting nuclear localization of EZH2, which in turn was identified to be responsible for endothelial inflammation through downregulation of KLF2 (Thakar et al., 2021). Although an increase in activating H3K4me3 and repressive H3K27me3 marking similar hyperglycemia treatment settings is unusual and interesting and how these epigenetic alterations represent a more dynamic bivalent chromatin state and, therefore, could regulate cell fate and differentiation is needed to be explored.

Epigenetic mechanisms are emerging as the key driver of EndMT (Alvandi and Bischoff, 2021). As our previous study indicates a possible role of EZH2 in diabetes-associated inflammatory switch of endothelial cells (Thakar et al., 2021), EZH2 indeed is also reported to be an important modulator of EndMT during both development and disease. During development of heart, EZH2 is recruited by HDAC3 to the DNA to mediate silencing of TGF-β1 transcription, thereby terminating physiological EndMT, which is an essential step to complete cardiac development (Lewandowski et al., 2015). A recent work describes that endothelial identity and function is critically controlled by the histone demethylase JMJD2B by regulating the level of H3K9me3, which is induced by EndMT-promoting, proinflammatory, and hypoxic conditions and supports acquiring a mesenchymal phenotype (Glaser et al., 2020). Acetyltransferase p300 expression was elevated in cardiac EC that are exposed to TGF-β2 treatment and regulated gene transcription of the histone acetyltransferase EP300 (E1A binding protein p300), which played an important role during EndMT (Ghosh et al., 2012). H4 (histone 4) acetylation through p300 was involved in the synergistic upregulation of specific SMAD3 target genes HEY1 and ankyrin repeat domain 1 on combined TGF-β1 stimulation and Notch activation, which in turn induced EndMT in EC (Fu et al., 2009). In fact, activation of Notch signaling pathways through a nitric oxide-sGC-dependent mechanism was responsible for early EndMT in the developing atrioventricular canal (Chang et al., 2011). Increased Notch activation in confluent human embryonic stem cells–EC monolayer cultures induces areas of EndMT containing transitional cells that are marked by increased Jagged1 expression (Eisenberg and Markwald, 1995). Herein, we observed activation of Notch signaling and the expression of Notch downstream gene Hes1, which in turn likely caused acquisition of partial mesenchymal character of EC.

Our previously published work (Thakar et al., 2021) reveals downregulation of KLF2 and KLF4 in HUVEC exposed to intermittent high glucose. KLF2 signaling was previously reported to play a crucial role in governing the EndMT (Dejana et al., 2017) phenotype. Through analysis of KLF2 and KLF4 in the present study, we found that both KLF2 and KLF4 are downregulated in HUVEC exposed to intermittent high glucose while attenuating the catalysis of H3K4me3 by using a pharmacological inhibitor or MLL2 siRNA rescued the level of KLF2 comparable to control while the KLF4 level in intermittent high-glucose-challenged HUVEC remains unaltered upon OCIR treatment. Although, in the present manuscript, we did not explore how alteration in MLL2-H3K4me3 regulates KLF2 expression, a relatively recent study indicates that MKL1 recruits an H3K4 trimethyltransferase SET1 to the promoter regions of p65 target genes to cause p65-associated gene expression in inflammatory settings (Yu et al., 2017). Because activation of p65 signaling causes suppression of KLF2 (Jha and Das, 2017), we, therefore, envisage that, in our present study, a high level of H3K4me3 upon intermittent high glucose likely caused expression of p65-associated genes, which further abrogated KLF2 expression, which is then neutralized upon normalization of intermittent high glucose-induced H3K4me3 level through OICR or MLL2 siRNA. However, such a hypothesis of KLF2 regulation through the MLL2-H3K4me3 axis still need to be experimentally validated in EC exposed to intermittent high glucose. In addition, many other signaling pathways in EC, including Hippo signaling (Zhang et al., 2014), Wnt-β-Catenin signaling (Liebner et al., 2004), and VEGF signaling (Paruchuri et al., 2006), govern the EndMT phenotype, which was not explored in the present study. Therefore, future experiments studying such pathways in current experimental settings will allow us to understand the signaling landscape of EC that caused acquisition of partial mesenchymal character upon intermittent high glucose exposure.

In our recently published work, we show that inflammatory stimuli in EC causes abrupt and dynamic changes in histone methylation, especially in H3K4me3 and H3K27me3 levels, which in turn causes de-repression of Notch ligand Jagged1, thereby promoting Notch activation and supporting endothelial inflammation (Katakia et al., 2021). In parallel to these observations, we identify that intermittent hyperglycemia exposure to EC causes abrupt enrichment of H3K4me3 on gene promoters of Notch ligands Jagged1 and Jagged2, which in turn causes their expression and hyperactivation of the Notch signaling pathway. In the present experimental settings, such hyperactivation of Notch1 signaling supports acquisition of partial mesenchymal character of EC, which was abrogated by administration of pharmacological inhibitors of MLL protein and siRNA mediated knockdown of MLL2. Such inhibition of MLL also caused deactivation of Notch signaling in intermittent hyperglycemia setting, which is likely responsible for reduced mesenchymal character in endothelial cells.

## Conclusion

In summary, intermittent high-glucose challenge to EC caused elevation in a specific SET1/COMPASS family of proteins, including MLL2 and WDR82, which promoted catalysis of H3K4me3. Enhanced H3K4me3 enriched the gene promoter region of Notch ligands Jagged1 and Jagged2, thereby causing their expression and concurrent Notch1 activation. Activation of Notch1 signaling through such a mechanism imparted partial mesenchymal character in EC and inhibiting the MLL class of methyltransferase caused Notch deactivation and revoked such partial mesenchymal transition of EC upon intermittent high-glucose exposure. An altered epigenetic state represented by an enhanced H3K4me3 level governed the maintenance of a fully differentiated state of EC upon hyperglycemia challenge, thus targeting such epigenetic pathways could be of potential interest in diabetes-associated cardiovascular complications.

## Data Availability

The original contributions presented in the study are included in the article/Supplementary Material, further inquiries can be directed to the corresponding author.

## Abbreviations

ADAM protein family, a disintegrin and metalloproteinase protein family; Dll1/4, Delta-like 1/4; EC, Endothelial cells; EndMT, Endothelial-to-mesenchymal transition; EZH2, Enhancer of zeste homolog 2; HGMEC, Human Glomerular Microvascular Endothelial Cells; H3K4me1/2/3, Histone 3 lysine 4 mono-/di-/ tri-methylation; HUVEC, Human umbilical vein endothelial cells; KLF2/4, Kruppel Like Factor 2/4; MLL1 (C), Mixed-lineage leukemia 1 C-terminal; MLL1 (N), Mixed-lineage leukemia 1 N-terminal; MLL2, Mixed-lineage leukemia 2 (KMT2D); N1-ICD, Notch1-intracellular domain; αSMA, α-smooth muscle actin; TGFβ, Transforming growth factor β; WDR5/82, WD Repeat Domain 5/82.
