# Supplementary material for: Elevated H3K4me3 Through MLL2-WDR82 upon Hyperglycemia Causes Jagged Ligand Dependent Notch Activation to Interplay with Differentiation State of Endothelial Cells
Source: Front Cell Dev Biol. 2022 Mar 22;10:839109. doi: 10.3389/fcell.2022.839109 (PMC8982561; doi:10.3389/fcell.2022.839109)
Supplement: Supplementary file 1 [file DataSheet2.pdf]

Supplemental Table 1. List of Antibodies

Antibody name	Catalogue no.
**Immunofluorescence cell studies**	
Alpha-Smooth Muscle Actin (D4K9N) XP(R) Antibody	19245 (CST)
Anti-VE-cadherin mouse antibody (F-8)	9989 (Santa Cruz)
Goat anti-Mouse Secondary Antibody, Alexa Fluor 488	A11001 (Thermo Fisher Scientific)
Goat anti-Rabbit Secondary Antibody, Alexa Fluor 555	A32732 (Thermo Fisher Scientific)
**mouse kidney immunofluorescence studies**	
CD31 Rabbit monoclonal antibody, 1:50	77699 (CST)
H3K4me3 Rabbit monoclonal antibody, 1:50	9751 (CST)
Alpha smooth muscle actin Rabbit polyclonal antibody, 1:200	ab5694 (Abcam)
Rhodamine Red-X-conjugated AffiniPure Fab Fragment Goat Anti-Rabbit	111-297-003 (Jackson Immunoresearch)
Alexa Fluor 488-conjugated AffiniPure Fab Fragment Goat Anti-Rabbit	111-547-003 (Jackson Immunoresearch)
**Immunoblotting studies**	
Cyclin D3 (DCS22) Mouse monoclonal antibody	2936 (CST)
HES1 (D6P2U) Rabbit monoclonal antibody	11988 (CST)
Cleaved Notch1 (Val1744) (D3B8) Rabbit monoclonal antibody	4147 (CST)
ADAM9 (D64B5) Rabbit monoclonal antibody	4151 (CST)
DLL4 Rabbit Polyclonal Antibody	2589 (CST)
DLL1 Rabbit Polyclonal Antibody	2588 (CST)
Jagged1 (28H8) Rabbit monoclonal antibody	2620 (CST)
Jagged2 (C23D2) Rabbit monoclonal antibody	2210 (CST)

TACE (D22H4) Rabbit monoclonal antibody	6978 (CST)
Tri-Methyl-Histone H3 (Lys4) (C42D8) Rabbit monoclonal antibody	9751 (CST)
Histone H3 (96C10) Mouse monoclonal antibody	3638 (CST)
CD31 (PECAM-1) (89C2) Mouse monoclonal antibody	3528 (CST)
Alpha-Smooth Muscle Actin (D4K9N) XP(R) Antibody	19245 (CST)
VE-Cadherin (D87F2) XP(R) Rabbit monoclonal antibody	2500 (CST)
Snail (C15D3) Rabbit monoclonal antibody	3879 (CST)
Slug (C19G7) Rabbit monoclonal antibody	9585 (CST)
SET1A (D3V9S) Rabbit monoclonal antibody	61702 (CST)
MLL1 (D2M7U) Rabbit monoclonal antibody (Amino-terminal Antigen)	14689 (CST)
MLL1 (D6G8N) Rabbit monoclonal antibody (Carboxy-terminal Antigen)	14197 (CST)
MLL2/KMT2B (D6X2E) Rabbit monoclonal antibody (Carboxy-terminal Antigen)	63735 (CST)
WDR82 (D2I3B) Rabbit monoclonal antibody	99715 (CST)
Menin (D45B1) XP® Rabbit monoclonal antibody	6891 (CST)
c-Myc (D84C12) Rabbit monoclonal antibody	5605 (CST)
Di-Methyl-Histone H3 (Lys4) (C64G9) Rabbit monoclonal antibody	9725 (CST)
Mono-Methyl-Histone H3 (Lys4) (D1A9) XP® Rabbit monoclonal antibody	5326 (CST)
KLF2 Rabbit Polyclonal Antibody	PA5-40591 (Thermo Fisher Scientific)
ADAM10 Rabbit Polyclonal Antibody	14194 (CST)
GAPDH (D16H11) XP® Rabbit monoclonal antibody	5174 (CST)

β-Tubulin (D3U1W) Mouse monoclonal antibody	86298 (CST)
KLF4 Rabbit Polyclonal antibody	4038 (CST)
N-Cadherin (D4R1H) XP® Rabbit monoclonal antibody	13116 (CST)
Vimentin (D21H3) XP® Rabbit monoclonal antibody	5741 (CST)

Supplemental Table 2. List of Primers used for quantification studies

Gene name	Forward sequence	Reverse sequence
Jagged 1 gene	*5`CCCCCTGTGAAGTGATTGAC3`*	*5`CCCGACTGACTCTTGCACTT3`*
Jagged 2 gene	*5`TGAAAAAACCTGATTGGCGG3`*	*5`CGACAGTCGTTGACGTTGAT3`*
Jagged 1 promoter	*5`GGCCAAAACTTTGTCCACCC3`*	*5`GGGCTTGCTGTTGCATTTGA3`*
Jagged 2 promoter	*5`GGGAGATTCTCGGAGGAGGT3`*	*5`CTCGCAGTCCCAGAGGAGAT3`*
Snai2 promoter	*5`ACAGTGTCCTGAAAGGGAGC3`*	*5`TAGCAAACGAAGCTGCGAGA3`*
ACTA2 promoter	*5`GGTTCTTAAGTGGGGGCCAT3`*	*5`GTGCAGTCGTTAGTGCGGTA3`*
Snai2 gene	*5`AACAGTATGTGCCTTGGGGG3`*	*5`AAAAGGCACTTGGAAGGGGT3`*
	5`GCTACCCAATGGCCTCTCTC3`	*5`CTTCAATGGCATGGGGGTCT3`*
FSP1 (S100A4) gene	*5`GACAGCAACAGGGACAACGA3`*	*5`TCAAGCACGTGTCTGAAGGA3`*
Vimentin gene	*5`AAACTTAGGGGCGCTCTTGT3`*	*5`GAGGGCTCCTAGCGGTTTAG3`*
Calponin (CNN1) gene	*5`GAGGTTAAGAACAAGCTGGCCC3`*	*5`GTTCTCAAACAGGTCGTTGGC3`*
Versican gene	*5`AGTGATGCGGGTCTTTACCG3`*	*5`GCGTCACACTGCTCAAATCC3`*
