# Supplementary material for: Elevated H3K4me3 Through MLL2-WDR82 upon Hyperglycemia Causes Jagged Ligand Dependent Notch Activation to Interplay with Differentiation State of Endothelial Cells
Source: Front Cell Dev Biol. 2022 Mar 22;10:839109. doi: 10.3389/fcell.2022.839109 (PMC8982561; doi:10.3389/fcell.2022.839109)
Supplement: Supplementary file 2 [file DataSheet1.pdf]

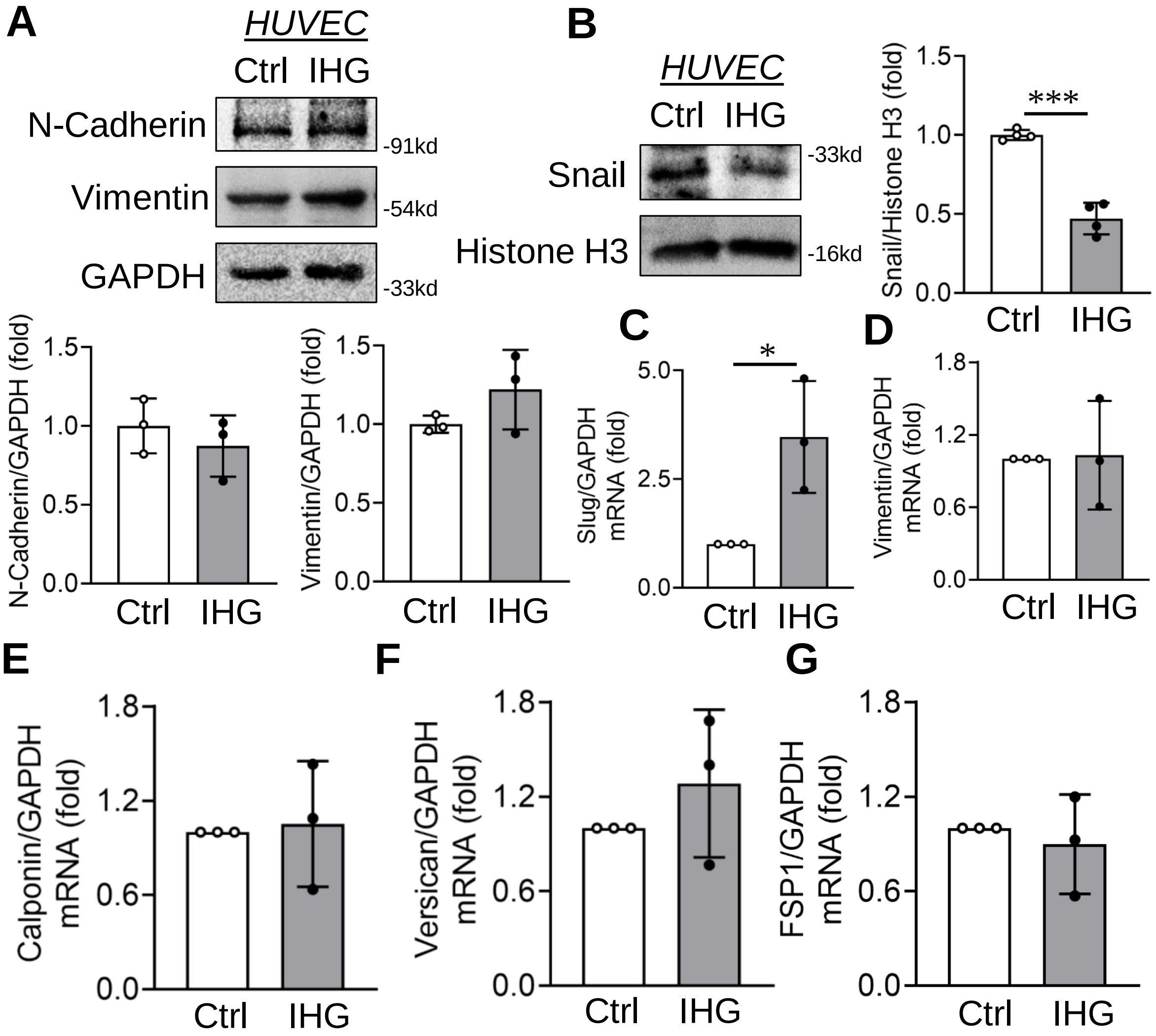

Supplemental Figure 1. Intermittent high glucose did not alter the level of many EndMT associated genes. (A-B) Immunoblot analysis of HUVEC exposed to intermittent high glucose followed measuring the level of N-Cadherin (A), Vimentin (A) and Snail (B). (C-G) Transcript level expression of genes; *Slug* (C), *Vimentin* (D), *Calponin* (E), *Versican* (F) and *FSP1* (G) in HUVEC challenged with intermittent high glucose. ***$p < 0.001$, by unpaired t-test.

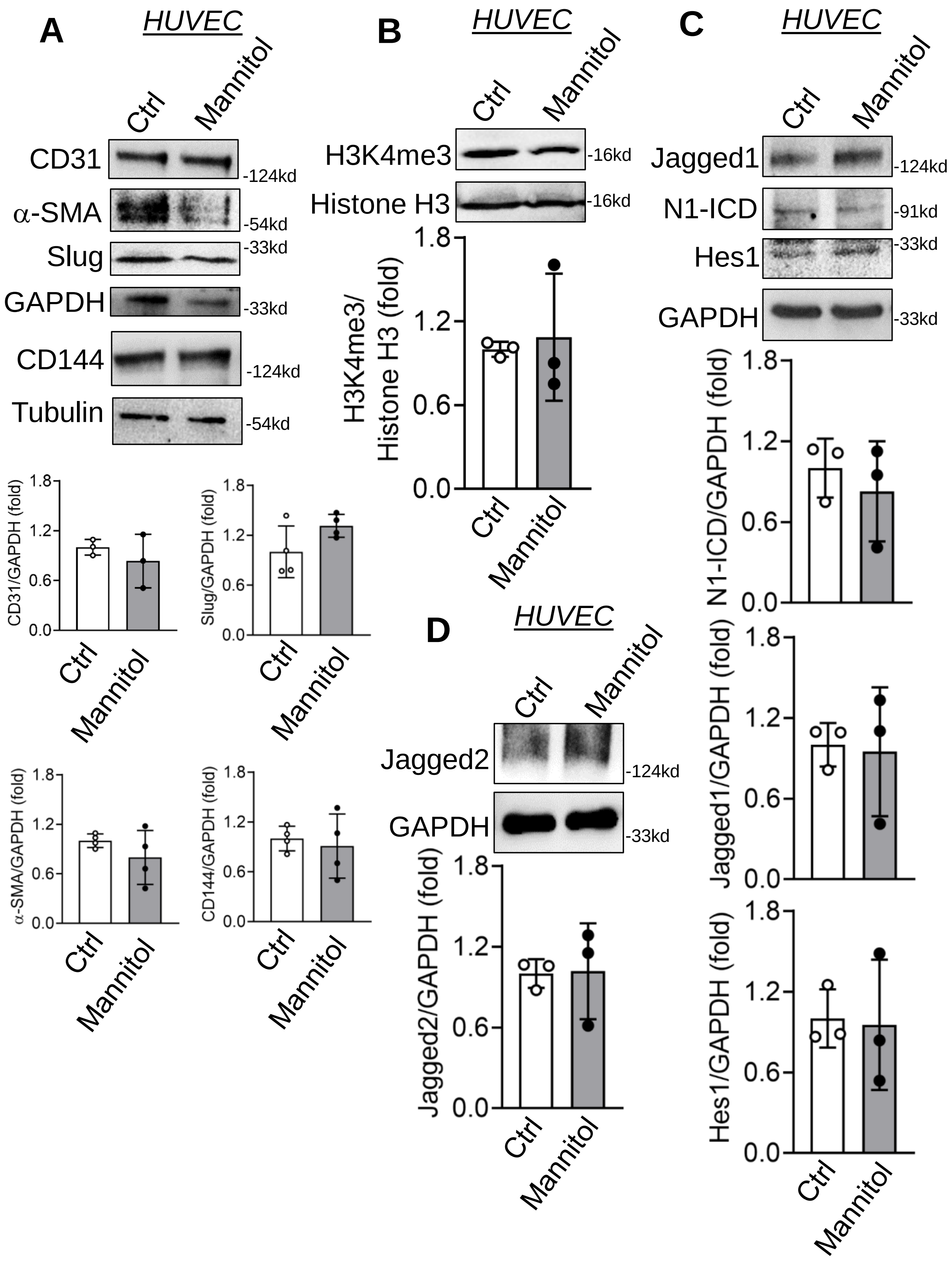
A
HUVEC
Ctrl
Mannitol
CD31
-124kd
α-SMA
-54kd
Slug
-33kd
GAPDH
-33kd
CD144
-124kd
Tubulin
-54kd
CD31/GAPDH (fold)
Slug/GAPDH (fold)
α-SMA/GAPDH (fold)
CD144/GAPDH (fold)
B
HUVEC
H3K4me3
-16kd
Histone H3
-16kd
H3K4me3/ Histone H3 (fold)
C
HUVEC
Jagged1
-124kd
N1-ICD
-91kd
Hes1
-33kd
GAPDH
-33kd
N1-ICD/GAPDH (fold)
Jagged1/GAPDH (fold)
Hes1/GAPDH (fold)
D
HUVEC
Jagged2
-124kd
GAPDH
-33kd
Jagged2/GAPDH (fold)
0.0
0.6
1.2
1.8

Supplemental Figure 2. Osmolality changes imparted through D-mannitol treatment do not alter EndMT and Notch signaling changes. (A) HUVEC were exposed to intermittent high glucose for three days followed by incubation for extended two days prior to assessing the level of CD31, α-SMA, Slug, CD144 through immunoblot analysis. (B-D) Immunoblot analysis of HUVEC lysates collected from cells treated with intermittent high mannitol treatment conditions (as comparable to glucose level) and probed for H3K4me3 (B), Jagged1 (C), N1-ICD (C), Hes1 (C), and Jagged2 (D) along with densitometry quantification.

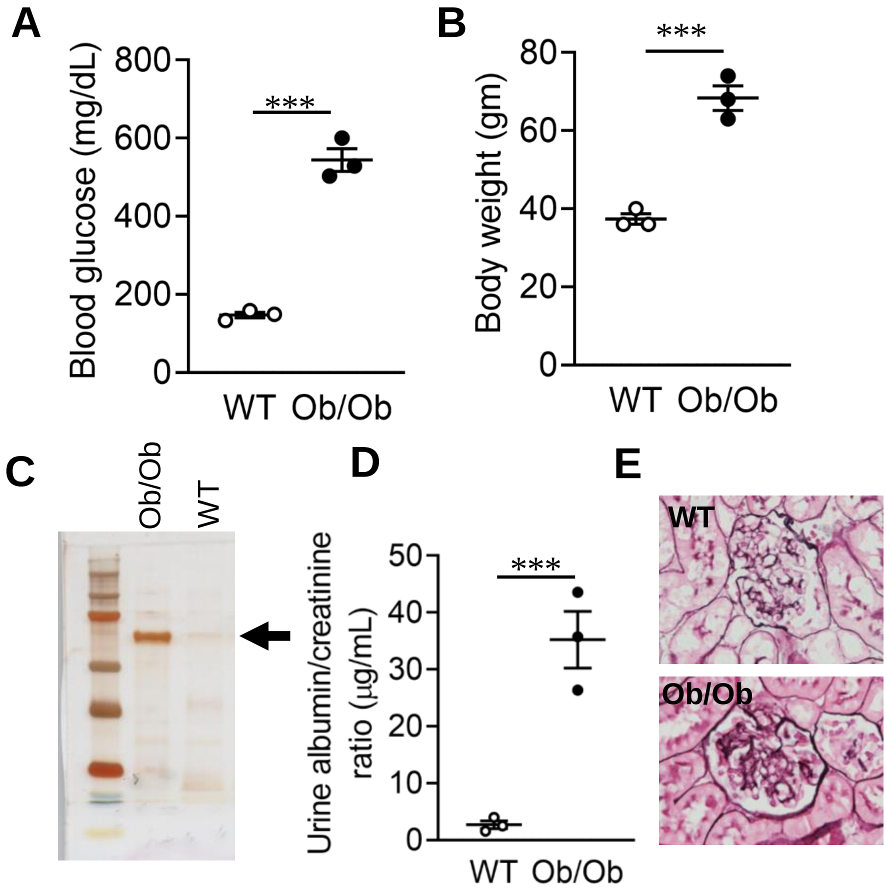

Supplemental Figure 3. Ob/Ob mice exhibited biochemical markers of glomerular dysfucntion. (A-B) Random blood glucose (A) and body weight (B) at 14 weeks of age of WT and Ob/Ob mice. (C) Silver stained SDS-PAGE gel of urine samples collected from 14 weeks old WT and Ob/Ob mice. (D) Albumin to Creatinine ratio (ACR) analysis of urine samples collected from male WT and Ob/Ob mice of 14 weeks of age. (E) Representative images of male WT and Ob/Ob mice kidney tissues stained with Periodic acid–Schiff and Silver stain. (n=3) ***$p < 0.001$, by unpaired t test.

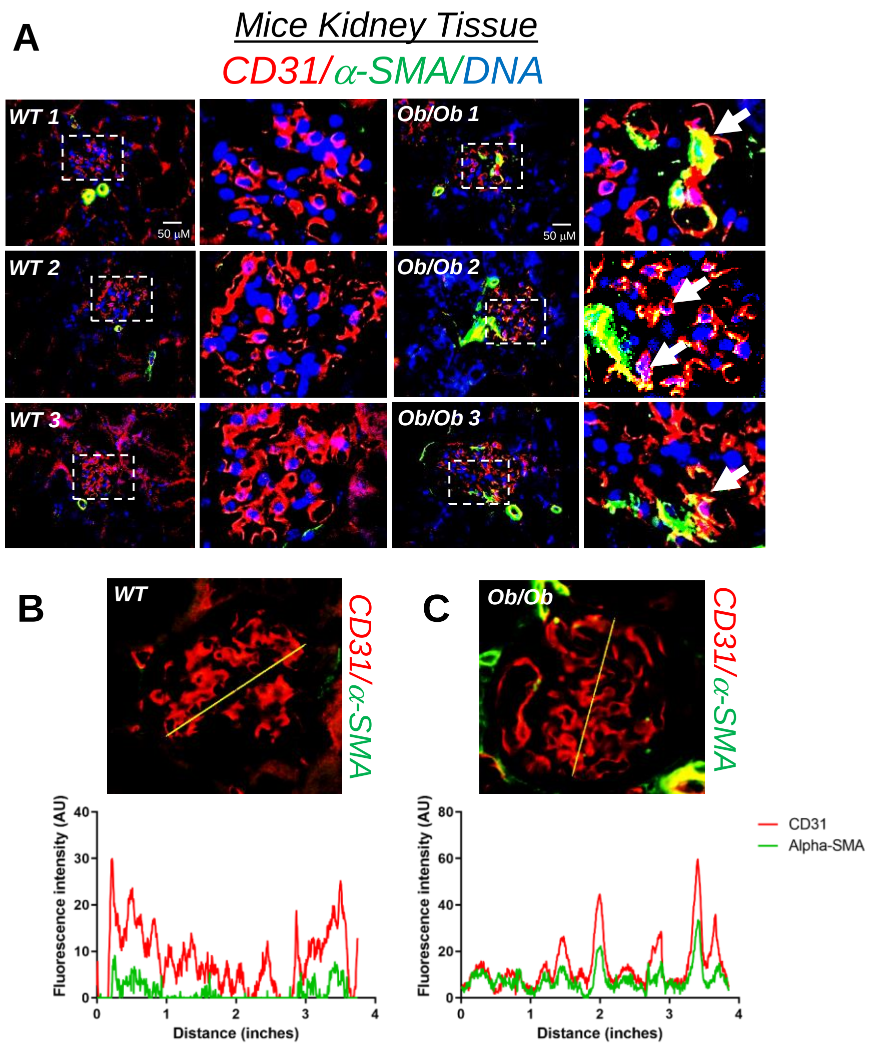

Supplemental Figure 4. Glomerular endothelial cells co-expressed CD31 and α-SMA in kidney section of Ob/Ob mice. (A) Immunohistochemistry of tissue sections from BTBR WT and BTBR Ob/Ob mice stained for α-SMA and CD31 (n=3). DAPI staining is shown in blue. White arrowheads indicate endothelial cells expressing both CD31 and α-SMA. (B-C) Line graph indicating pixel-wise intensities of green and red fluorescence in the yellow line highlighted areas within the image.

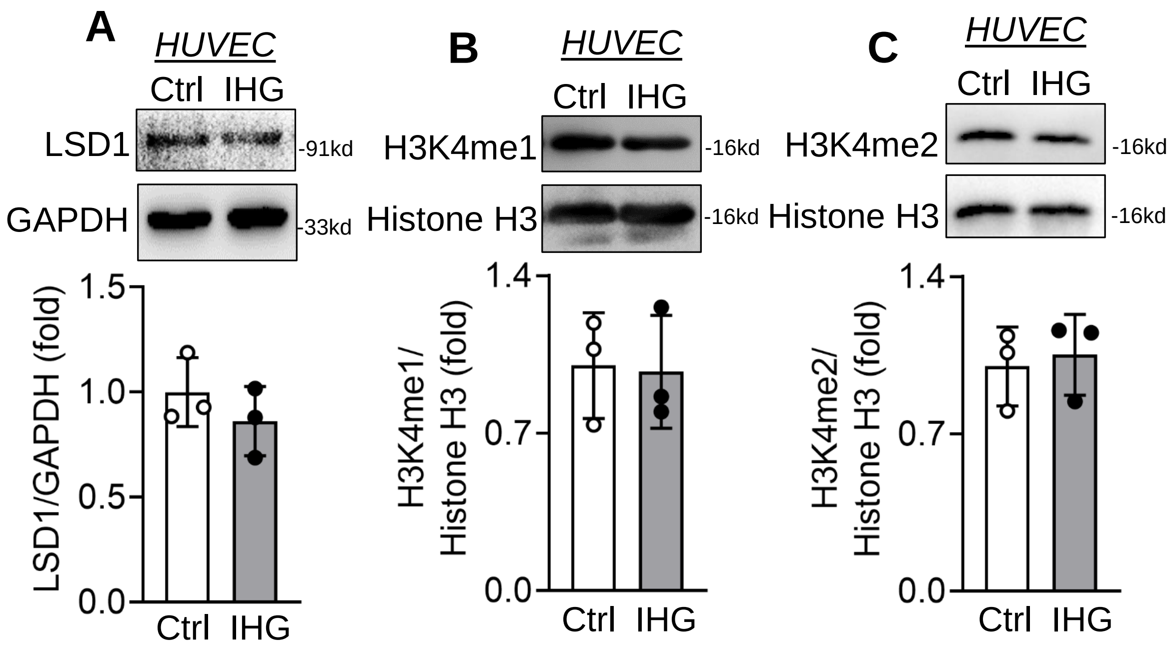

Supplemental Figure 5. Intermittent hyperglycemia do not alter H3K4 associated demethylase or its downstream targets H3K4me1 and H3K4me2. (A-C) Immunoblot analysis of HUVEC lysates collected from cells treated with intermittent high glucose treatment conditions and probed for Lsd1 (A), H3K4me1 (B), and H3K4me2 (C) along with densitometry quantification.

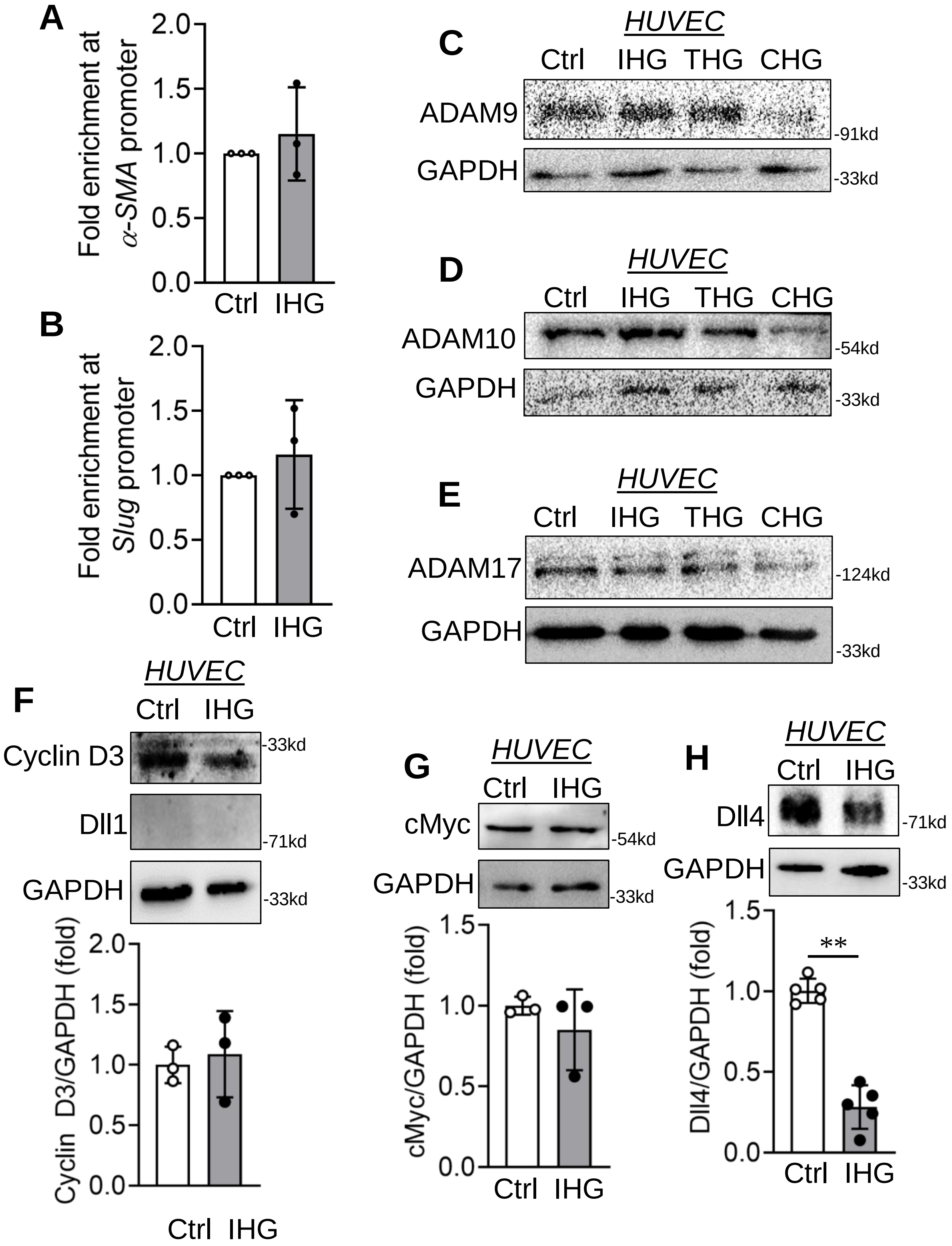

Supplemental Figure 6. Intermittent high glucose exposure induced H3K4me3 did not enrich into *α-SMA* and *Slug* gene promoters as well as did not alter the expression of Notch-

associated ADAM-family of metalloproteinase while reducing the expression of Notch ligands Dll4. (A-B) ChIP-qPCR analysis of H3K4me3 enrichment on *α-SMA* (A, n=3) and *Slug* (B, n=3) gene promoters in HUVEC exposed to intermittent hyperglycemia. (C-E) Immunoblot analysis of endothelial cell lysates collected from HUVEC treated with intermittent, transient, and constant high glucose and probed for ADAM9 (C), ADAM10 (D), ADAM17 (E). (F-H) Immunoblot analysis of HUVEC lysates collected from cells exposed to intermittent high glucose treatment conditions and probed for Cyclin D3 (F), Dll1 (F), cMyc (H), and Dll4 (G). $^{**}p < 0.01$, by unpaired t test.

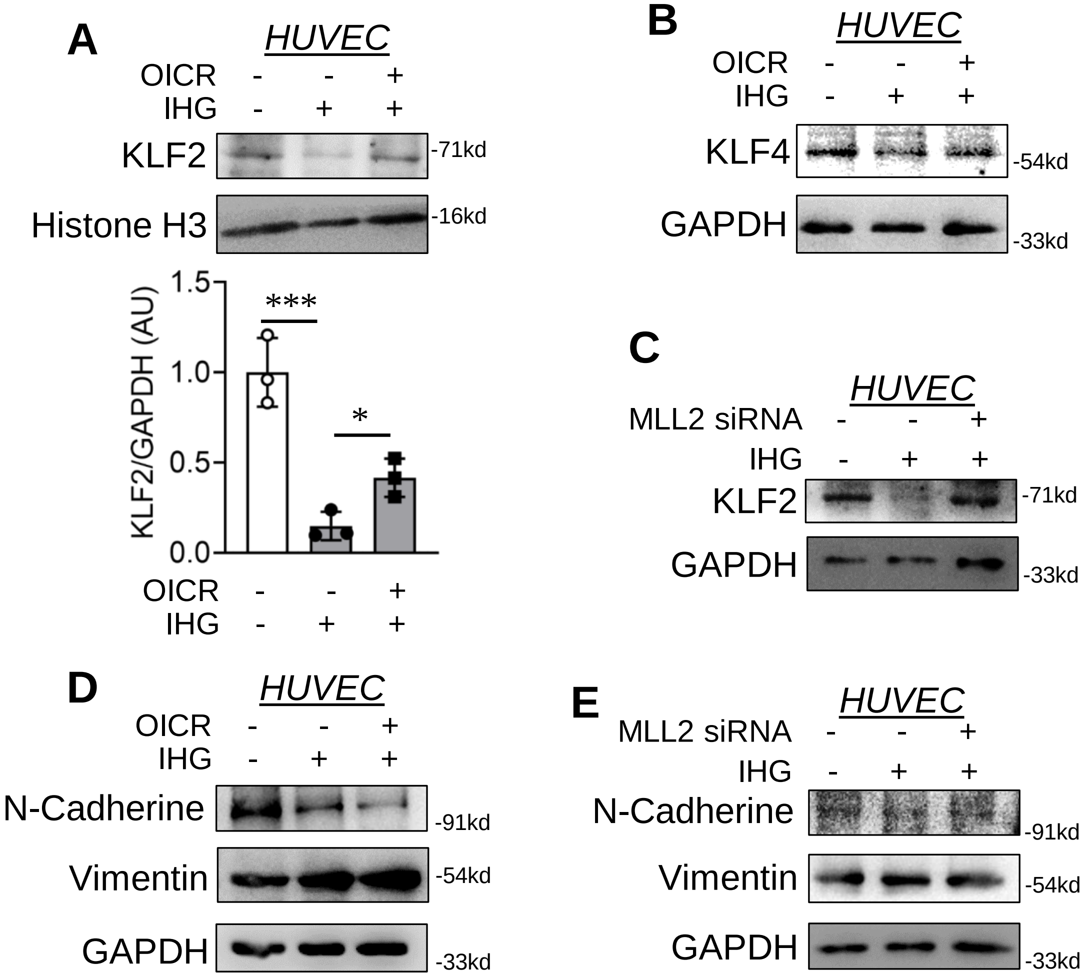

Supplemental Figure 7. Catalytic inhibition or siRNA-mediated knock down of MLL blocked intermittent high glucose dependent reduction in KLF2 level without much altering KLF4. (A-E) Immunoblot analysis of endothelial cell lysates collected from HUVEC treated with intermittent high glucose and OICR (A,B,D) and/or MLL2 siRNA (C,E) probed for KLF2 (A,C), KLF4 (B), N-Cadherin and vimentin (D-E)). HUVEC in which KLF2 and KLF4 were detected cells were exposed to three days of intermittent high glucose exposure while cells whereas for cells where N-cadherin and vimentin were detected, HUVEC are exposed to five days treatment conditions. *$p<0.05$ and ***$p < 0.001$, by Two-stage linear step-up procedure of Benjamin, Krieger and Yekutieli test.
